# Test battery for measuring the perception and recognition of facial expressions of emotion

**DOI:** 10.3389/fpsyg.2014.00404

**Published:** 2014-05-13

**Authors:** Oliver Wilhelm, Andrea Hildebrandt, Karsten Manske, Annekathrin Schacht, Werner Sommer

**Affiliations:** ^1^Department of Psychology, Ulm UniversityUlm, Germany; ^2^Department of Psychology, Humboldt-Universität zu BerlinBerlin, Germany; ^3^CRC Text Structures, University of GöttingenGöttingen, Germany

**Keywords:** emotion perception, emotion recognition, individual differences, psychometrics, facial expression

## Abstract

Despite the importance of perceiving and recognizing facial expressions in everyday life, there is no comprehensive test battery for the multivariate assessment of these abilities. As a first step toward such a compilation, we present 16 tasks that measure the perception and recognition of facial emotion expressions, and data illustrating each task's difficulty and reliability. The scoring of these tasks focuses on either the speed or accuracy of performance. A sample of 269 healthy young adults completed all tasks. In general, accuracy and reaction time measures for emotion-general scores showed acceptable and high estimates of internal consistency and factor reliability. Emotion-specific scores yielded lower reliabilities, yet high enough to encourage further studies with such measures. Analyses of task difficulty revealed that all tasks are suitable for measuring emotion perception and emotion recognition related abilities in normal populations.

## Introduction

Facial expressions are indispensable sources of information in face-to-face communication (e.g., Todorov, 2011). Thus, a crucial component of successful personal interactions is to rapidly perceive facial expressions and correctly infer others' internal states they convey. Although there is on-going debate between emotion theorists about the functions, and meanings of facial expressions, most contemporary approaches propose that facial expressions of emotions are determined by evaluation results and represent the efferent effects of the latter on motor behavior (cf. Scherer et al., 2013). Specific facial expressions are emotions of the person the face belongs to (Walla and Panksepp, 2013) and they play a crucial role in emotion communication. The perception and identification of emotions from faces predicts performance on socio-emotional measures and peer ratings of socio-emotional skills (e.g., Elfenbein and Ambady, 2002a; Rubin et al., 2005; Bommer et al., 2009). These measures of socio-emotional competences include task demands that ask the participant to perceive emotional facial expressions (e.g., Mayer et al., 1999). However, the measurement of emotional abilities should also include mnestic tasks because facial expressions of emotions in face-to-face interactions are often short-lived and the judgment of persons may partly rely on retrieval of their previous facial expressions. Attempts to measure the recognition of previously memorized expressions of emotions are rare. We will discuss available tasks for measuring emotion perception and recognition from faces and point to some shortcomings regarding psychometrics and task applicability. After this brief review, we will suggest theoretical and psychometric criteria for a novel task battery designed to measure the accuracy and the speed of performance in perceiving and recognizing emotion in faces.

## The assessment of emotion perception and recognition from faces

The most frequently used task for measuring the perception and identification of emotions from faces is the *Brief Affect Recognition Test* (BART; Ekman and Friesen, 1974) and its enhanced version, the *Japanese and Caucasian Brief Affective Recognition Test* (JACBART; Matsumoto et al., 2000). The drawback of these tasks is that they present stimuli for a limited time (presentations last only two seconds) and therefore stress perceptual speed arguably more than measures avoiding such strictly timed stimulus expositions (e.g., Hildebrandt et al., 2012). However, if stimuli of the BART or JACBART were presented for longer durations, performance on these tasks in unimpaired subjects would show a ceiling effect because most adults recognize prototypical expressions of the six basic emotions with high confidence and accuracy (Izard, 1971).

The *Diagnostic Analysis of Nonverbal Accuracy* (DANVA; Nowicki and Carton, 1993) is frequently used in individual difference research (e.g., Elfenbein and Ambady, 2002a; Mayer et al., 2008). The DANVA stimuli are faces of adults and children displaying one of four emotional expressions (happiness, sadness, anger, and fear) that vary between pictures in their intensity levels with the use of variable intensity levels corresponding to item difficulty. Therefore, the test can provide adequate performance scores for emotion recognition ability across a broad range of facial characteristics but it relies on a single assessment method (affect naming). Stimulus exposition in the DANVA is also speeded. Thus, it is unclear to what extent individual differences in performance are due to the difficulty of recognizing expressions of emotions of lower intensity and to what extent they are due to the speeded nature of the task.

Stimuli for the frequently used *Profile of Nonverbal Sensitivity* (PONS; Rosenthal et al., 1979) includes faces, voices, and body images and assesses emotion recognition from multimodal and dynamic stimuli. The use of dynamic facial stimuli is a positive feature of PONS because it ensures a more naturalistic setting. One drawback is that it is limited to two emotion categories (positive vs. negative affect) and only one method of affect naming.

The *Multimodal Emotion Recognition Test* (MERT; Bänziger et al., 2009) has the virtue of using both static and dynamic facial stimuli for testing the recognition of 10 emotion expression categories. However, MERT is limited by implementing labeling as the sole method of affect naming.

In a recent publication, Palermo et al. (2013) presented two novel tests (tasks) developed with the aim of overcoming some of the problems of the aforementioned tasks. One task presented by Palermo and colleagues was inspired by Herzmann et al. (2008) and implemented the odd-man-out paradigm with the aim of measuring the perception of facial expressions of emotion. The second task is based on the frequently used labeling paradigm that captures emotion identification and naming emotion expressions. Both tasks are based on stimuli presenting six categories of emotional expressions (happiness, surprise, fear, sadness, disgust, and anger) and were shown to capture individual differences in performance accuracy; thus, accuracy rates showed no ceiling effect in an unimpaired sample of 80 adults with an age range of 18–49 years. A further virtue of the test is its two-method approach. The test described by Palermo et al. is a first step for developing a multivariate task battery for measuring emotion perception and identification in non-clinical samples.

Further task batteries have been described and used in the neuropsychological literature and were mainly developed for clinical purposes. Some examples are the *Florida Affect Battery* (FAB; Bowers et al., 1989) and the *Comprehensive Affect Testing System* (CATS; Froming et al., 2006). The FAB uses facial, vocal, and cross-modal stimuli and multiple methods (discrimination, naming, selection, and matching); but it uses only static face stimuli. The multiple modalities and multiple methods approach of the FAB are outstanding features, but an important drawback of FAB is the low difficulty for unimpaired subjects (Bowers et al., 1989). This limitation also applies to the CATS.

The revised *Reading the Mind in the Eye* Test (Baron-Cohen et al., 2001), originally intended to measure social sensitivity, arguably captures emotion recognition from the eye area only. In a four-alternative forced-choice-decision paradigm, stimuli depicting static eye regions are used that are assumed to represent 36 complex mental states (e.g., tentative, hostile, decisive, etc.). Target response categories and their foils are of the same valence to increase item difficulty. This test aims to measure individual differences in recognizing complex affective states (Vellante et al., 2012), however the test is not appropriate to capture individual differences in the perception of emotions because there are no unequivocal and veridical solutions for the items.

Finally, there is a series of experimental paradigms designed to measure the identification of emotion in faces (e.g., Kessler et al., 2002; Montagne et al., 2007). Single task approaches have the disadvantage that the measured performance variance due to a specific assessment method cannot be accounted for when assessing ability scores. Below we will mention experimental paradigms that were a source of inspiration for us in the process of task development when we describe the task battery.

The literature about mnemonic emotional face tasks is sparse as compared with the abundance of emotion identification paradigms. In the memory task described by Hoheisel and Kryspin-Exner (2005)—the *Vienna Memory of Emotion Recognition Tasks* (VIEMER)—participants are presented with a series of faces showing emotional expressions. The participants' task is to memorize the facial identities for later recall. Individual faces presented with an emotional expression during the learning period are then displayed during the later recall period including several target and distracter faces all showing neutral expressions. Participants must identify the faces that were seen earlier during the learning period with an emotional expression. This task does not measure emotion recognition *per se* but the interplay of identity and expression recognition and does not allow for statistical measurement of method variance. Similarly, experimental research on memory for emotional faces (e.g., D'Argembeau and Van der Linden, 2004; Grady et al., 2007) aimed to investigate the effects of emotional expressions on face identity recognition. This measure, in addition to the VIEMER, reflects an unknown mixture of expression and identity recognition. Next, we will briefly describe the criteria that guided our development of emotion perception and recognition tasks. After theoretical considerations for the test construction, we will outline psychometric issues.

## Theories and models

The perception and identification of facially expressed emotions has been described as one of the basic abilities located at the lowest level of a hierarchical taxonomic model of Emotional Intelligence (e.g., Mayer et al., 2008). The mechanisms underlying the processing of facial identity and expression information and their neural correlates have been widely investigated in the neuro-cognitive literature. Models of face processing (Bruce and Young, 1986; Calder and Young, 2005; Haxby and Gobbini, 2011) delineate stages of processing involved in recognizing two classes of facial information: (1) *pictorial aspects* and invariant *facial structures* that code facial identity and allow for extracting person-related knowledge at later processing stages; and (2) *changeable aspects* that provide information for action and emotion understanding (most prominently eye gaze and facial expressions of emotion). In their original model, Bruce and Young (1986) suggested that at an initial stage of structural encoding, during which view-centered descriptions are constructed from the retinal input, the face processing stream separates into two pathways—one being involved in identifying the person and the second involved in processing changeable facial information such as facial expression or lip speech. Calder (2011) reviewed evidence from image-based analyses of faces, experimental effects representing similar configural and holistic processing of identity and facial expressions, but also neuroimaging and neuropsychological data. He concluded that at a perceptual stage there seems to be a partly common processing route for identity and expression-related facial information (see also Young and Bruce, 2011). Herzmann et al. (2008) published a comprehensive task battery for the *multivariate* measurement of face cognition abilities—that is, of identity-related facial information processing. The present work aims to complement that task battery with measures assessing the ability to perceive and recognize facial emotion expressions—that is, abilities regarding the processing of variable facial characteristics.

Prevalent measures of emotion expression perception rely on classifying prototypical expression stimuli into emotion categories. Under conditions of unlimited time, unimpaired subjects frequently perform at ceiling in such tasks. In order to avoid such ceiling effects researchers frequently manipulate task difficulty by using brief exposition times for stimuli. Such manipulations, if done properly, will decrease accuracy rates as desired—but they do not eliminate speed-related individual differences. Limited exposition times are likely to favor participants high in perceptual speed. Difficulty manipulations based on psychological theory (e.g., using composites of emotion expressions in stimuli, manipulate intensity of emotion expression) are conceptually better suited for developing novel measures of individual differences in unimpaired populations.

Following functional models of facial emotion processing, we define *perception of facial emotion expression* as the ability to visually analyze the configuration of facial muscle orientations and movements in order to identify the emotion to which a particular expression is most similar. Based upon a well-established distinction in intelligence research (Carroll, 1993) we seek to distinguish between measures challenging the accuracy and the speed of performance, respectively. Speed measures of emotion perception are designed to capture the swiftness of decisions about facial emotion expressions and the identification of the emotion of which they are associated. Accuracy measures of emotion perception should assess the correctness of emotion identification. We define *memory for facial emotion expressions* as the ability to correctly encode, store, and retrieve emotional expressions from long-term memory. Speeded memory tasks are easy recognition tasks that capture the time required to correctly recognize previously well-learned emotion expressions. Accuracy based memory tasks express the degree to which previously learned emotional faces, that were not over learned, are correctly identified during recall.

## Desiderata for task development and psychometric considerations

A *first* crucial requirement on test construction is to base the measurement intention on models of the neuro-cognitive processes that ought to be measured. *Second*, an integrative view incorporating experimental and individual difference evidence is facilitated if the developed experimental task paradigms are adapted to psychometric needs (O'Sullivan, 2007; Scherer, 2007; Wilhelm et al., 2010). Without reliance on basic emotion research there is considerable arbitrariness in deriving tasks from broad construct definitions. *Third*, scores from single tasks are inadequate manifestations of highly general dispositions. This problem is prevalent in experimental approaches to studying emotion processing. Other things being equal, a multivariate approach to measure cognitive abilities is generally superior to task specific measures because it allows for abstracting from task specificities. *Fourth,* assessment tools should be based on a broad and profoundly understood stimulus base. Wilhelm (2005) pointed to several measurement specificities that are commonly treated as irrelevant in measuring emotional abilities. Specifically, generalizations from a very restricted number of stimuli are a neglected concern (for a more general discussion, see Judd et al., 2012). O'Sullivan (2007) emphasized the impact of a profound understanding of stimulus characteristics for measuring emotional abilities and conjectured that this understanding is inadequate for most of the available measures.

The following presented work describes conceptual and psychometric features of a multivariate test battery. We assessed the difficulty and the psychometric quality of a broad variety of performance indicators that can be derived on the basis of 16 tasks for measuring the accuracy or speed of the perception or recognition of facially expressed emotions. Then, the battery will be evaluated and applied in subsequent studies. All tasks are available for non-commercial research purposes upon request from the corresponding author.

## Methods

### Sample

A total of 273 young adults (who reported to have no psychiatric disorders), between 18 and 35 years of age, participated in the study. They all lived in the Berlin area and self-identified as Caucasian. Participants were contacted via newspaper advertisements, posters, flyers, and databases of potential participants. Due to technical problems and dropouts between testing sessions, four participants had missing values for more than five tasks and were excluded from the analyses. The final sample included 269 participants (52% females). Their mean age was 26 years (*SD* = 6). Their educational background was heterogeneous: 26.8% did not have degrees qualifying for college education, 62.5% had only high school degrees, and 10.7% held academic degrees (i.e., some sort of college education). All participants had normal or corrected-to-normal visual acuity.

### Development of the emotional face stimuli database used for the tasks

#### Photo shooting

Pictures were taken in individual photo sessions with 145 (72 males) Caucasian adults ranging in age from 18 to 35 years. Models were recruited via newspaper advertisements. Photographs were taken with similar lighting and identical background conditions. Models did not wear makeup, piercings, or beards. Glasses were removed during the shooting and when needed, hair was fixed outside the facial area. In order to elicit emotional expressions, we followed the procedure described by Ebner et al. (2010). Each photography session consisted of three phases: emotion induction, personal experiences, and imitation. Photographs were taken continuously through all three phases, and at least 150 pictures of each person were stored. Each exposure was taken from three perspectives (frontal and right and left three-quarter views) with synchronized cameras (Nikon D-SLR, D5000) from a distance of 3 meters. From this pool of 145 faces, 122 face identities (50% females) in total were used as stimuli across all tasks. The pictures were selected according to their photographic quality and expression quality evaluated by a trained researcher and FaceReader software codes (see validation below).

Expression elicitation was structured into three phases; the three phases were completed for one emotion before starting with the production of the next emotional expression. The sequence was: neutral, sadness, disgust, fear, happiness, anger, and surprise. The first expression elicitation phase was the *Emotion Induction Phase*. We elicited emotional expressions by a subset of 16 pictures from the International Affective Picture System (IAPS; Lang et al., 2008) that were presented one after another by being projected on a back wall. Models were asked to carefully look at the pictures, identify which emotion the picture elicited in them, and display that emotion in their face with the intention to communicate it spontaneously. Models were also instructed to communicate the emotion with their face at a level of intensity that would make a person not seeing the stimulus picture understand what emotion the picture elicited in them. We used four neutral, five sadness, six disgust, seven fear, and four happiness inducing IAPS pictures. Since there are no IAPS pictures for anger, the induction phase for anger started with the second phase. After all pictures within one emotion were presented continuously, models were asked to choose one of the pictures for closer inspection. During the inspection of the selected picture further photographs were taken.

The second expression elicitation phase was the *Personal Experience Phase.* Models were asked to imagine a personally relevant episode of their lives in which they strongly experienced a certain emotional state corresponding to one of the six emotions (happiness, surprise, fear, sadness, disgust, and anger). The instructions were the same as in the first phase: communicate an emotional expression with the face so that a second person would understand the expressed feeling.

The third expression elicitation phase was the *Imitation Phase.* Models were instructed by written and spoken instructions based on emotion descriptions according to Ekman and Friesen (1976) regarding how to exert the specific muscular activities required for expressing the six emotions in the face. In contrast to the previous phases, no emotional involvement was necessary in the imitation part. Models were guided to focus on the relevant areas around the eyes, the nose, and the mouth and instructed on how to activate these regions in order to specifically express one of the six basic emotions. During this phase photographers continuously supported the models by providing them with feedback. Models were also requested to inspect their facial expression in a mirror. They had the opportunity to compare their own expression with the presented expression model from the database by Ekman and Friesen (1976) and to synchronize their expression with a projected prototypical emotion portrait.

#### Validation of facial expressions

First, trained researchers and student assistants selected those pictures that had an acceptable photographic quality. From all selected pictures those that clearly expressed the intended emotion, including low intensity pictures, were selected for all models. Facial expressions were coded regarding the target emotional expression along with the other five basic emotion expressions and neutral expressions using FaceReader 3 software (http://www.noldus.com/webfm_send/569). Based on FaceReader 3 emotion ratings, the stimuli were assigned to the tasks described below. Overall accuracy rate of FaceReader 3 at classifying expressions of younger adults is estimated 0.89 and classification performance for separate emotion categories are as follows: Happiness 0.97; Surprise 0.85; Fear 0.93; Sadness 0.85; Disgust 0.88; and Anger 0.80 (Den Uyl and van Kuilenburg, 2005).

#### Editing

All final portraits were converted to grayscale and fitted with a standardized head-size into a vertical elliptical frame of 200 × 300 pixels. During post-processing of the images, differences in skin texture were adjusted and non-facial cues, like ears, hair and clothing, were eliminated. Physical attributes like luminance and contrast were held constant across images. Each task was balanced with an equal number of female and male stimuli. Whenever two different identities were simultaneously presented in a given trial, portraits of same sex models were used.

### General procedure

All tasks were administered by trained proctors in group-sessions with up to 10 participants. There were three sessions for every participant, each lasting about three hours, including two breaks of 10 min. Sessions were completed in approximately weekly intervals. Both task and trial sequences were kept constant across all participants. Computers with 17-inch monitors (screen definition: 1366 × 768 pixel; refresh rate: 60 Hz) were used for task administration. The tasks were programmed in Inquisit 3.2 (Millisecond Software). Each task started at the same time for all participants in a given group. In general, participants were asked to work to the best of their ability as quickly as possible. They were instructed to use the left and right index fingers during tasks that used two response options and to keep the fingers positioned directly above the relevant keys throughout the whole task. Tasks with four response options were organized such that the participant only used the index finger of a preferred hand. Every single task was introduced by proctors and additional instructions were provide on screen. There were short practice blocks in each task consisting of at least 5 and at most 10 trials (depending on task difficulty) with trial-by-trial feedback about accuracy. There was no feedback for any of the test trials. Table 1 gives an overview of the tasks included in the task battery.

**Table 1:** **Overview of the tasks**.

**Task**	**Name of the task**	**Ability domain**	**Duration in min.**	**# of Blocks/Trials**	**# of Faces**
1	Identification of emotion expressions from composite faces	EP	10	1/72	8
2	Identification of emotion expressions of different intensity from upright and inverted dynamic face stimuli	EP	12	1/72	12
3	Visual search for faces with corresponding emotion expressions of different intensity	EP	17	1/40	4
4	Emotion hexagon—identification of mix-ratios in expression continua	EP	15	1/60	10
5	Emotion hexagon—discrimination	EP	10	1/60	10
6	Learning and recognition of emotion expressions of different intensity	EM	18	4/72	4
7	Learning and recognition of emotional expressions from different viewpoints	EM	15	4/56	4
8	Learning and recognition of mixed emotion expressions in expression morphs	EM	15	4/56	4
9	Cued emotional expressions span	EM	10	7/32	4
10	Memory for facial expressions of emotions	EM	10	4/27	4
11	Emotion perception from different viewpoints	SoEP	8	1/31	14
12	Emotional odd-man-out	SoEP	6	1/30	18
13	Identification speed of emotional expressions	SoEP	10	1/48	8
14	*1*-back recognition speed of emotional expressions	SoEM	8	4/24	4
15	Delayed non-matching to sample with emotional expressions	SoEM	10	1/36	18
16	Recognition speed of morphed emotional expressions	SoEM	6	6/36	6

EP, emotion perception; EM, emotion memory; SoEP, speed of emotion perception; SoEM, speed of emotion memory; the expected predominant source of performance variability is the performance accuracy for the tasks 1–10 and the response latency (speed) for the tasks 11–16; Duration in min. includes instruction time and practice; # of Blocks/Trials: number of blocks and the sum of trials across block included in the task; # of Faces: number of face identities used to design a specific task; the total number of face identities used in the task battery is 145; the total duration is 180 min.

### Data treatment and statistical analyses

The final dataset (*N* = 269) was visually screened for outliers in uni- and bivariate distributions. Outliers in univariate distributions were set to missing. For the approximately 0.2% of missing values after outlier elimination a multiple random imputation (e.g., Allison, 2001) was conducted. With this procedure, plausible values were computed as predicted values for missing observations plus a random draw from the residual normal distribution of the respective variable. One of the multiple datasets was used for the analyses reported here. Results were verified and do not differ from datasets obtained through multiple imputation with the **R** package *mice,* by van Buuren and Groothuis-Oudshoorn (2011).

Reaction time (RT) scores were only computed from correct responses. RTs smaller than 200 ms were set to missing, because they were considered too short to represent proper processing. The remaining RTs were winsorized (e.g., Barnett and Lewis, 1978); that is, RTs longer than 3 *SD*s above the individual mean were fixed to the individual mean RT plus 3 *SD*. This procedure was repeated iteratively beginning with the slowest response until there were no more RTs above the criterion of 3 *SD*.

All analyses were conducted with the statistical software environment **R**. Repeated measures ANOVAs (rmANOVA) were performed with the package *ez* (Lawrence, 2011) and reliability estimates with the package *psych* (Revelle, 2013).

### Scoring

For accuracy tasks, we defined the proportion of correctly solved trials of an experimental condition of interest (e.g., emotion category, expression intensity, presentation mode) as the performance indicator. For some of these tasks we applied additional scoring procedures as indicated in the corresponding task description. Speed indicators were average inverted RTs (measures in seconds) obtained across all correct responses associated with the trials from the experimental conditions of interest. Note that accuracy was expected to be at ceiling in measures of speed. Inverted latency was calculated as 1000 divided by the RT in milliseconds.

## Perception and identification tasks of facial emotion expressions

### Task 1: identification of emotion expressions from composite faces

Calder et al. (2000) proposed the *Composite Face Paradigm* (e.g., Young et al., 1987) for investigating perceptual mechanisms underlying facial expression processing and particularly for studying the role of configural information in expression perception. Composite facial expressions were created by aligning the upper and the lower face half of the same person, but from photos with different emotional expressions, so that in the final photo each face was expressing an emotion in the upper half of the face that differed from the emotion expressed in the lower half of the face. Aligned face halves of incongruent expressions lead to holistic interference.

**Figure 1:**
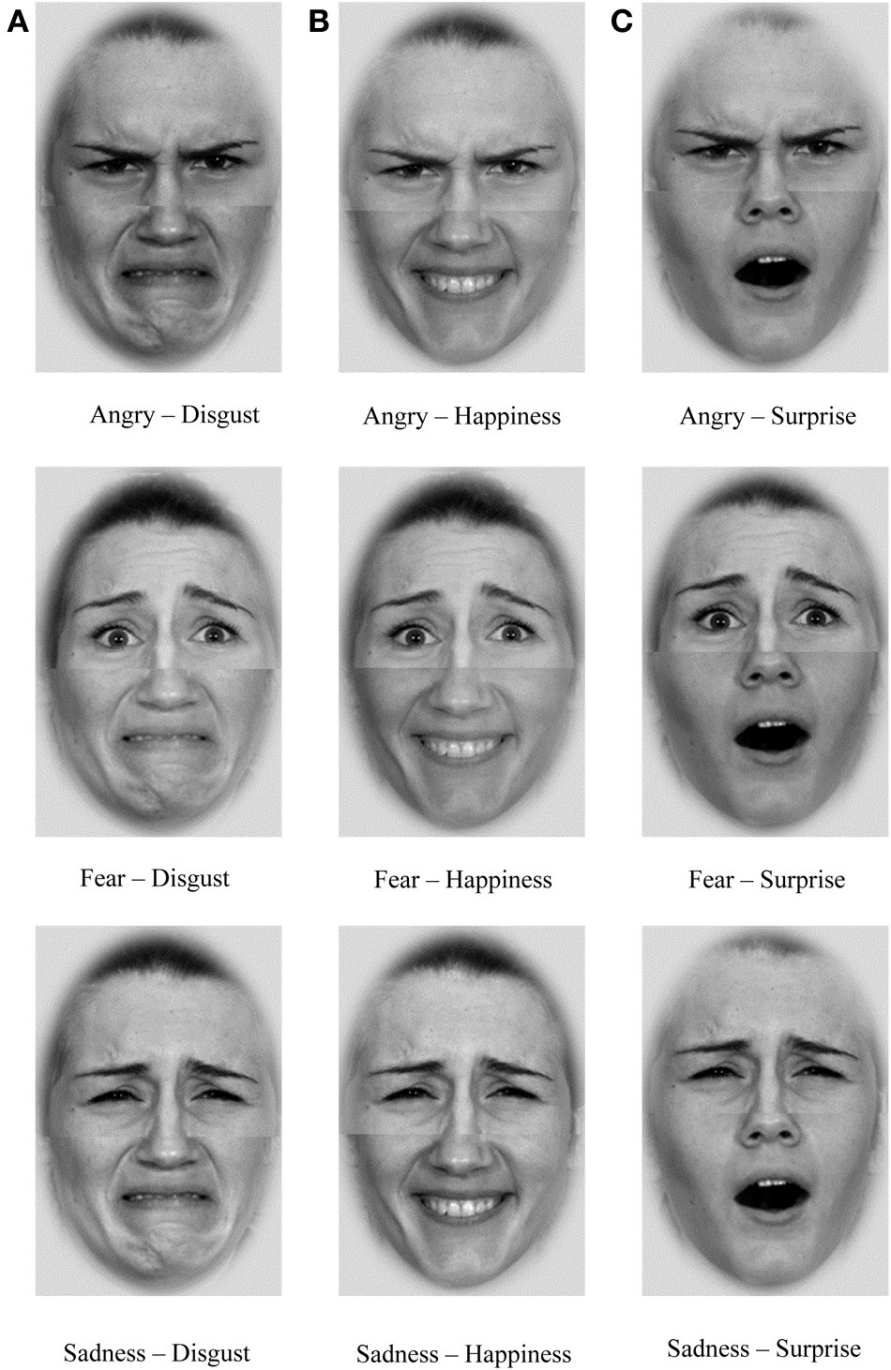
**Stimuli examples used in Task 1 (Identification of emotion expression from composite faces)**.

It has been shown that an emotion expressed in only one face half is less accurately recognized compared to congruent emotional expressions in face composites (e.g., Tanaka et al., 2012). In order to avoid ceiling effects, as is common for the perception of emotions from prototypical expressions, we took advantage of the higher task difficulty imposed by combining different facial expressions in the top and bottom halves of faces, and exploited the differential importance of the top and bottom face for the recognition of specific emotions (Ekman et al., 1972; Bassili, 1979). Specifically, fear, sadness, and anger are more readily recognized in the top half of the face and happiness, surprise, and disgust in the bottom half of the face (Calder et al., 2000). Here, we used the more readily recognizable halves for the target halves in order to ensure acceptable performance. Top halves expressing fear, sadness, or anger were only combined with bottom halves expressing disgust, happiness, or surprise—yielding nine different composites (see Figure 1 for examples of all possible composite expression stimuli of a female model).

#### Procedure

After the instruction and nine practice trials, 72 experimental trials were administered. The trial sequence was random across the nine different emotion composites. Pictures with emotional expressions of four female and four male models were used to create the 72 emotion composites. For each model, nine aligned composite faces were created. In each trial, following a fixation cross, a composite face was presented in the center of the screen. The prompt words “TOP” or “BOTTOM” were shown above the composite face to indicate for which face half the expression should be classified; the other half of the face was to be ignored. Six labeled “buttons” (from left to right: “happiness,” “surprise,” “anger,” “fear,” “sadness,” “disgust”) were aligned in a horizontal row on the screen below the stimuli. Participants were asked to click with a computer mouse the button corresponding to the emotion in the prompted face half. After the button was clicked the face disappeared and the screen remained blank for 500 ms; then the next trial started with the fixation cross.

#### Scoring

In addition to the proportion of correct responses across a series of 72 trials, we calculated unbiased hit rates (*H_u_*; Wagner, 1993). Unbiased hit rates account for response biases toward a specific category and correct for systematic confusions between emotion categories. For a specific emotion score *H_u_* was calculated as squared frequency of the correct classifications divided by the product of the number of stimuli used for the different emotion categories and the overall frequency of choices for the target emotion category. We report difficulty estimates for both percent correct and *H_u_*.

#### Results and discussion

Table 2 summarizes performance accuracy across all administered trials and for specific emotional expressions along with reliability estimates computed with Cronbach's Alpha (α) and Omega (ω; McDonald, 1999). We calculated reliabilities on the basis of percent correct scores. Difficulty estimates in Table 2 based on percent correct scores show that performance was not at ceiling. The distributions across persons for the happiness, surprise, and anger trials were negatively skewed (−1.61, −0.87, −1.05), suggesting a somewhat censored distributions to the right, but for no participant was accuracy at ceiling. In an rmANOVA the emotion category showed a strong main effect: [*F*_(5, 1340)_ = 224.40, *p* < 0.001, η^2^ = 0.36]. *Post-hoc* analyses indicate happiness was recognized the best, followed by surprise, anger, disgust, fear, and sadness. This ranking was similar for *H_u_* scores (see Table 2). However, when response biases were controlled for, anger was recognized better than surprise. Percent correct and *H_u_* scores across all trials were correlated 0.99 (*p* < 0.001), indicating that the scoring procedure do not notably affect the rank order of persons.

**Table 2:** **Descriptive statistics and reliability estimates of performance accuracy for all emotion perception tasks across all trials and for single target emotions**.

**Condition**	**Accuracy *M* (*SD, SE*)**	**Alternative score *M* (*SD, SE*)**	**Alpha / Omega / # of trials**
**TASK 1: IDENTIFICATION OF EMOTION EXPRESSIONS FROM COMPOSITE FACES**			
Overall (range: 0.37–0.89)	0.66 (0.11, 0.01)	0.47 (0.14, 0.01)^**^	0.81/0.81/72
Happiness (1^*^)	0.84 (0.19, 0.01)	0.59 (0.19, 0.01)	0.74/0.75/12
Surprise (2)	0.78 (0.20, 0.01)	0.51 (0.19, 0.01)	0.73/0.73/12
Fear (5)	0.49 (0.20, 0.01)	0.30 (0.17, 0.01)	0.61/0.61/12
Sadness (6)	0.45 (0.22, 0.01)	0.31 (0.19, 0.01)	0.69/0.70/12
Disgust (4)	0.66 (0.19, 0.01)	0.51 (0.21, 0.01)	0.67/0.67/12
Anger (3)	0.76 (0.17, 0.01)	0.59 (0.20, 0.01)	0.59/0.59/12
**TASK 2: IDENTIFICATION OF EMOTION EXPRESSIONS FROM UPRIGHT AND INVERTED DYNAMIC FACES**			
Overall (range: 0.46–0.85)	0.68 (0.07, 0.00)	0.48 (0.09, 0.01)^**^	0.62/0.62/72
Happiness (1^*^)	0.94 (0.07, 0.00)	0.76 (0.13, 0.01)	0.23/0.32/09^***^
Surprise (2)	0.83 (0.13, 0.01)	0.59 (0.13, 0.01)	0.61/0.63/12
Fear (6)	0.49 (0.20, 0.01)	0.31 (0.17, 0.01)	0.61/0.62/13
Sadness (5)	0.55 (0.19, 0.01)	0.40 (0.15, 0.01)	0.55/0.56/12
Disgust (3)	0.67 (0.19, 0.01)	0.43 (0.16, 0.01)	0.65/0.65/12
Anger (4)	0.63 (0.14, 0.01)	0.42 (0.12, 0.01)	0.29/0.31/11
**TASK 3: VISUAL SEARCH FOR FACES WITH CORRESPONDING EMOTION EXPRESSIONS**			
Overall (range: 0.24–0.94)	0.76 (0.14, 0.01)	4.54 (0.70, 0.04)	0.86/0.87/40
Surprise (1^*^)	0.89 (0.15, 0.01)	5.42 (0.87, 0.05)	0.60/0.61/08
Fear (5)	0.60 (0.22, 0.01)	4.03 (1.07, 0.07)	0.47/0.48/08
Sadness (3)	0.82 (0.17, 0.01)	5.00 (0.91, 0.06)	0.62/0.63/08
Disgust (2)	0.86 (0.19, 0.01)	5.42 (0.95, 0.06)	0.64/0.64/08
Anger (4)	0.62 (0.22, 0.01)	3.41 (0.94, 0.06)	0.53/0.54/08
**TASK 4: EMOTION HEXAGON—IDENTIFICATION OF MIX-RATIOS IN EXPRESSION CONTINUA**			
Overall (range: 8.26–60.51)	14.29 (5.38, 0.33)^****^	–	0.93/0.94/60
Happiness (1^*^)	11.67 (5.96, 0.36)	–	0.78/0.80/10
Surprise (5)	15.36 (5.67, 0.35)	–	0.66/0.69/10
Fear (4)	15.27 (6.35, 0.39)	–	0.63/0.66/10
Sadness (6)	16.82 (5.89, 0.36)	–	0.61/0.64/10
Disgust (3)	14.15 (6.03, 0.37)	–	0.69/0.71/10
Anger (2)	12.48 (6.47, 0.39)	–	0.75/0.78/10
**TASK 5: EMOTION HEXAGON—DISCRIMINATION**			
Overall (range: 0.62–0.92)	0.80 (0.06, 0.00)	–	0.63/0.64/60
Happiness (1^*^)	0.90 (0.11, 0.01)	–	0.39/0.44/10
Surprise (3)	0.78 (0.13, 0.01)	–	0.24/0.26/10
Fear (2)	0.81 (0.12, 0.01)	–	0.27/0.28/10
Sadness (5)	0.64 (0.16, 0.01)	–	0.33/0.35/10
Disgust (1)	0.90 (0.10, 0.01)	–	0.13/0.21/10
Anger (4)	0.76 (0.15, 0.01)	–	0.45/0.47/10

Note. M, means, SE, standard errors, SD, standard deviations; Alpha (α) and Omega (ω) coefficients; # of Trials, the number of trials used for calculating the reliability coefficients; task names are shortened for this table;

* , the rank order of recognizability across emotions is indicated in the brackets;

** , Unbiased Hit Rate (Wagner, 1993);

*** , there was no variance in three items because they have been correctly solved by all subjects, reliability estimates are based on 9 out of 12 trials displaying facial expressions of happiness;

**** , score: amount of deviation of participants' response from the correct proportion of the mixture between the two parent expressions; the chance probability in case of Task 1 and 2 is 0.16 and 0.50 for Task 5; the chance probability is no relevant measure for Task 3 and 4.

Reliability estimates across all trials were very good and across all trials for a single emotion, considering the low number of trials for single emotions and the unavoidable heterogeneity of facial stimuli, were satisfactory (ranging between 0.59 and 0.75). Difficulty estimates suggest that performance across persons was not at ceiling. The psychometric quality of single emotion expression scores and performance on the overall measure are satisfactory to high. Adding more trials to the task could further increase the reliability of the emotion specific performance indicators.

### Task 2: identification of emotion expressions of different intensity from upright and inverted dynamic faces

Motion facilitates emotion recognition from faces (e.g., Wehrle et al., 2000; Recio et al., 2011). Kamachi et al. (2001) used morphed videos simulating the dynamics of emotion expressions and showed that they are partly encoded on the basis of static information but also from motion-related cues. Ambadar et al. (2005) demonstrated that facial motion also promotes the identification accuracy of subtle, less intense emotion displays. In Task 2, we used dynamic stimuli in order to extend the measurement of emotion identification to more real life-like situations and to ensure adequate construct representation of the final task battery (Embretson, 1983).

Because previous findings predict higher accuracy rates for emotion identification from dynamic stimuli, we implemented intensity manipulations in order to avoid ceiling effects. Hess et al. (1997) investigated whether the intensity of a facial emotion expression is a function of muscle displacement compared with a neutral expression and reported decreased accuracy rates for static expression morphs of lower expression intensity. We generated expression-end-states by morphing intermediate expressions between a neutral and an emotional face. Mixture ratios for the morphs aimed at three intensity levels by decreasing the proportion of neutral relative to the full emotion expressions from 60:40% (low intensity) to 40:60% (middle) to 20:80% (high intensity).

In order to capture the contrast between configural vs. feature-based processing of facial expressions, we also included stimulus orientation manipulations (upright vs. inverted). Face inversion strongly impedes holistic processing, allowing mainly feature-based processing (Calder et al., 2000). McKelvie (1995) indicated an increase of errors and RTs of emotion perception from static faces presented upside-down and similar findings were reported for dynamic stimuli as well (Ambadar et al., 2005).

#### Procedure

Short videos (picture size was 200 × 300 pixel) displaying 30 frames per second were presented in the center of the screen. The first frame of the video displayed a neutral facial expression that, across the subsequent frames, changed to an emotional facial expression. The videos ended at 500 ms and the peak expression displayed in the last frame remained on the screen until the categorization was performed. Emotion label buttons were the same as in the previous task. We varied expression intensity across trials, with one third of the trials for each intensity level. The morphing procedure was similar to the procedure used in previous studies (e.g., Kessler et al., 2005; Montagne et al., 2007; Hoffmann et al., 2010) and included two steps. First, static pictures were generated by morphing a neutral expression image of a face model with the images of the same person showing one of the 6 basic emotions; mixture ratios were 40, 60, or 80 percent of the emotional face. Second, short video sequences were produced on the basis of a morphed sequence of frames starting from a neutral expression and ending with one of emotional faces generated in the first step. Thus, video sequences were created for all three intensities; this was done separately for two female and two male models. Half of the 72 trials were presented upright and the other presented upside down. Following the instructions participants completed four practice trials. The experimental trials with varying conditions (upright vs. upside-down), basic emotion, and intensity were presented in pseudo-randomized order but fixed across participants.

#### Results and discussion

**Figure 2:**
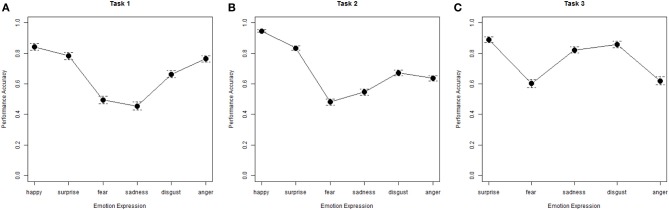
**Plots of the rank order of recognizability of the different emotion categories esteemed in emotion perception task. (A)** Task 1, Identification of Emotion Expression from composite faces; **(B)** Task 2, Identification of Emotion Expression of different intensity from upright and inverted dynamic face stimuli; **(C)** Task 3, Visual search for faces with corresponding Emotion Expression of different intensity, error bars represent confidence intervals.

In addition to results for the percent correct scores, we also report unbiased hit rates (see above). Table 2 summarizes the average performance calculated for both, percent correct and unbiased hit rates (the scores are correlated 0.98) along with reliability estimates, which were all acceptable, except the low omega for anger recognition. It seems that the facial expressions of anger used here were particularly heterogeneous. There were no ceiling effects in any of the indicators. An rmANOVA with factors for emotion expression and expression intensity revealed main effects for both. Emotion expression explained 34% of the variance in recognition rates, [*F*_(5, 1340)_ = 327.87, *p* < 0.001, η^2^ = 0.34] whereas the intensity effect was small [*F*_(2, 536)_ = 17.98, *p* < 0.001, η^2^ = 0.01]. The rank order of recognizability of different emotional expressions was comparable with Task 1, which used expression composites (cf. Figures 2A,B). Happiness and surprise were recognized the best, followed by anger and disgust, and finally sadness and fear were the most difficult. An interaction of emotion expression and intensity, [*F*_(10, 2680)_ = 96.94, *p* < 0.001, η^2^ = 0.13], may indicate that expression peaks of face prototypes used for morphing varied in their intensity between models and emotions. Scores calculated across all trials within single emotions disregarding the intensity manipulation had acceptable or good psychometric quality.

### Task 3: visual search for faces with corresponding emotion expressions of different intensity

Task 3 was inspired by the visual search paradigm often implemented for investigating attention biases to emotional faces (e.g., Frischen et al., 2008). In general, visual search tasks require the identification of a target object that differs in at least one feature (e.g., orientation, distance, color, or content) from non-target objects displayed at the same time. In this task, participants had to recognize several target facial expressions that differed from a prevailing emotion expression. Usually, reaction time slopes are inspected as dependent performance variables in visual search tasks. However, we set no limits on response time and encouraged participants to screen and correct their responses before confirming their choice. This way we aimed to minimize the influence of visual saliency of different emotions on the search efficiency due to pre-attentive processes (Calvo and Nummenmaa, 2008) and capture intentional processing instead. This task assessed the ability to discriminate between different emotional facial expressions.

#### Procedure

**Figure 3:**
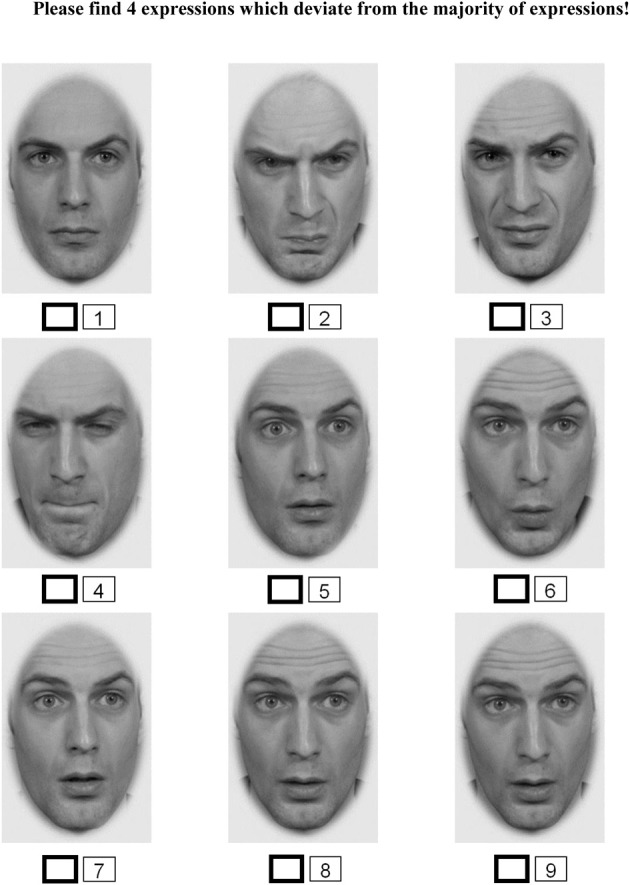
**Schematic representation of a trail from Task 3 (Visual search for faces with corresponding emotion expression of different intensity)**.

In each trial, a set of nine images of the same identity was presented simultaneously, arranged in a 3 × 3 grid. The majority of the images displayed one emotional expression (surprise, fear, sadness, disgust, or anger) referred to here as the *target expression*. In each trial participants were asked to identify the neutral and emotional expressions. Experimental manipulations incorporated in each trial were: (1) choice of distracter emotion expression, (2) the number of distracter emotion expressions—ranging from 1 to 4, and (3) the target expression. Happiness expressions were not used in this task because performance for smiling faces was assumed to be at ceiling due to pop out effects. The location of target stimuli within the grid was pseudo-randomized. Reminders at the top of the screen informed participants of the number of distracters to be detected in a given trial (see Figure 3 for an example). Participants' task was to identify and indicate all distracter expressions by clicking with their mouse a tick box below each stimulus. It was possible to review and correct all answers before submitting one's response; participants confirmed their responses by clicking the “next” button starting the next trial. The task aimed to implement two levels of difficulty by using target and distracter expressions with low and high intensity.

All 360 stimuli were different images originating from four models (two females and two males). Intensity level was assessed with the *FaceReader* software. Based on these intensity levels, trials were composed of either low or high intense emotion stimuli for targets as well as for distracters within the same trial. The number of divergent expressions to be identified was distributed uniformly across conditions. There were 40 experimental trials administered after three practice trials, which followed the instructions. The accuracies of the multiple answers for a trial are dependent variables.

#### Scoring

We applied three different scoring procedures. The first was based on the proportion of correctly recognized targets. This procedure only accounts for the hit rates, disregards false alarms, and can be used to evaluate the detection rate of target facial expressions. For the second, we computed a difference score between the hit-rate and false-alarm rate for each trial. This score is an indicator of the ability to recognize distracter expressions. For the third, we calculated d'prime-scores [*Z*(hit rate) − *Z*(false-alarm rate)] for each trial separately. The average correlation between the three scores was *r* = 0.96 (*p* < 0.001), suggesting that the rank order of individual differences was practically invariant across scoring procedures. Next, we will report proportion correct scores. Table 2 additionally displays average performance based on the d'prime scores.

#### Results and discussion

The univariate distributions of emotion-specific performance indicators and the average performance—displayed in Table 2—suggest substantial individual differences in accuracy measures. The task design was successful at avoiding ceiling effects frequently observed for recognition performance of prototypical expressions. This was presumably achieved by using stimuli of varying expression intensity and by the increasing number of distracters across trials. Reliability estimates of the overall score were excellent (α = 0.86; ω = 0.87). Considering that only eight trials entered the emotion specific scores and that emotional expressions are rather heterogeneous, reliability estimates (ranging from 0.48–0.64) are satisfactory.

An rmANOVA with two within subject factors, emotional expression and difficulty (high vs. low expression intensity), revealed that the expressed emotion explained 21% of the variance of recognition rates, [*F*_(4, 1072)_ = 244.86, *p* < 0.001, η^2^ = 0.21]. The rank orders of recognizability of the emotion categories were slightly different from those estimated in Task 1 and 2 (see Figures 2C,B compared with Figures 2A,B). Surprised faces were recognized the best, as was the case for Task 2. Anger faces were recognized considerably worse than sadness faces. This inconsistency might be due to effects of stimulus sampling. Performance on fear expressions was the poorest.

The difficulty manipulation based on high vs. low intensity of the target emotional expression, as well as intensity of distracter expressions, was successful as expressed by the main effect of difficulty in the rmANOVA, [*F*_(1, 268)_ = 638.26, *p* < 0.001, η^2^ = 0.16]. There was a significant interaction between intensity and emotion category [*F*_(4, 1072)_ = 100.82, *p* < 0.001, η^2^ = 0.09], were more intense expressions were recognized better within each expression category but to a different degree. The ratios of the difference between low and high intensity conditions varied across emotions: surprise—*M*_easy_ = 0.93, *M*_difficult_ = 0.84 [*t*_(268)_ = 8.05, *p* < 0.001]; fear—*M*_easy_ = 0.83, *M*_difficult_ = 0.37 [*t*_(268)_ = 21.96, *p* < 0.001]; sadness—*M*_easy_ = 0.88, *M*_difficult_ = 0.76 [*t*_(268)_ = 9.81, *p* < 0.001]; disgust—*M*_easy_ = 0.89, *M*_difficult_ = 0.82 [*t*_(268)_ = 4.62, *p* < 0.001]; and anger—*M*_easy_ = 0.77, *M*_difficult_ = 0.45 [*t*_(268)_ = 13.93, *p* < 0.001]. We conclude that performance indicators derived from this task have acceptable psychometric quality. Empirical difficulty levels differ across the intended manipulations based on expression intensity and the task revealed a rank order of recognizability similar to other tasks used in this study. The scoring procedure hardly affected the rank order of persons, allowing the conclusion that different scores derived from this task express the same emotional expression discrimination ability.

### Task 4: emotion hexagon—identification of mix-ratios in expression continua

It is suggested that the encoding of facial emotion expressions is based on discrete categorical (qualitative) matching (Etcoff and Magee, 1992; Calder et al., 1996), but also on the multidimensional perception of continuous information (Russell, 1980). There is evidence that both types of perception are integrated and used complementary (Fujimura et al., 2012). In this task, we required participants to determine the mixture ratios of two prototypical expressions of emotions. In order to avoid memory-related processes we constructed a simultaneous matching task. We morphed expressions of two emotions along a continuum of 10 mixture ratios. We only morphed continua between adjacent emotions on a so-called emotion hexagon (with the sequence happiness-surprise-fear-sadness-disgust-anger), where proximity of emotions represents potentially stronger confusion between expressions (e.g., Calder et al., 1996; Sprengelmeyer et al., 1996). In terms of categorical perception, there should be an advantage in identifying the correct mixture-ratio at the end of a continuum compared with more balanced stimuli in the middle of the continuum between two expression categories (Calder et al., 1996).

#### Procedure

Morphed images were created from two different expressions with theoretically postulated and empirically tested maximal confusion rates (Ekman and Friesen, 1976). Thus, morphs were created on the following six continua: happiness–surprise, surprise–fear, fear–sadness, sadness–disgust, disgust–anger, and anger–happiness. The mixture ratios were composed in 10% steps from 95:5 to 5:95. These morphs were created for each face separately for five female and five male models.

In every trial, two images of the same identity were presented on the upper left and on the upper right side of the screen, where each image displayed a different prototypical emotion expression (happiness, surprise, fear, sadness, disgust, and anger). Below these faces, centered on the screen, was a single expression morphed from the prototypical faces displayed in the upper part of the screen. All three faces remained on the screen until participants responded. Participants were asked to estimate the ratio of the morphed photo on a continuous visual analog scale. Participants were then instructed that the left and right ends of the scale represent a 100% agreement respectively with the images presented in the upper left and upper right side of the screen, and the middle of the scale represents a proportion of 50:50 from both parent faces. Participants were asked to estimate the mixture-ratio of the morph photo as exactly as possible, using the full range of the scale. There were no time limits. Three practice trials preceded 60 experimental trials. We scored performance accuracy as the average absolute deviation of participants' response from the correct proportion of the mixture between the two parent expressions.

#### Results and discussion

Table 2 displays the average overall and emotion specific deviation scores. An rmANOVA revealed that the emotion combinations used in this task were less influential than in other tasks, [*F*_(5, 1340)_ = 106.27, *p* < 0.001, η^2^ = 0.08]. Reliability estimates were excellent for the overall score (α = 0.93; ω = 0.94) and satisfactory for emotion-specific scores (ω ranged between 0.64 and 0.80). Further, it was interesting to investigate whether performance was higher toward the ends of the continua as predicted by categorical accounts of emotional expression perception. An rmANOVA with the within-subject factor mixture ratio (levels: 95, 85, 75, 65, and 55% of the prevailing emotional expression) showed a significant effect, [*F*_(4, 1072)_ = 85.27, *p* < 0.001, η^2^ = 0.13]. As expected, deviation scores were lowest at mixture ratios of 95% of a parent expression and increased with decreasing contributions of the prevailing emotion: *M*_95%_ = 9.04, *M*_85%_ = 13.33, *M*_75%_ = 15.89, *M*_65%_ = 17.11, *M*_55%_ = 16.09. There was no significant difference between the mixture levels 75, 65, and 55% of the target parent expression. A series of two-tailed paired *t*-tests compared differences between the emotion categories of the parent photo. The correct mixture ratio was better identified in the following combinations: performance in happiness with surprise combinations was slightly better than combinations of happiness with anger, [*t*_(268)_ = 1.78, *p* = 0.08]; surprise with happiness was easier to identify than surprise with fear, [*t*_(268)_ = 12.23, *p* < 0.001]; fear with sadness better than with surprise, [*t*_(268)_ = 9.67, *p* < 0.001]; disgust with sadness better than with anger, [*t*_(268)_ = 7.93, *p* < 0.001]; and anger with happiness better than with disgust, [*t*_(268)_ = 4.06, *p* < 0.001]. For sadness there was no difference between fear and disgust, [*t*_(268)_ = 0.37, *p* = 0.36]. Generally, we expected mixtures of more similar expressions to bias the evaluation of the morphs. The results are essentially in line with these expectations based on expression similarities.

Taken together, the results suggest the deviation scores meet psychometric standards. Performance improved or worsened as predicted by theories of categorical perception. Future research should examine whether expression assignment in morphed emotions is indicative of the ability to identify prototypical emotion expressions.

### Task 5: emotion hexagon—discrimination

This task is a forced choice version of the previously described Task 4 and aims to measure categorical perception of emotional expressions using a further assessment method.

#### Procedure

Participants were asked to decide whether the morphed expression presented in the upper middle of the screen was more similar to the expression prototype displayed on the lower left or lower right side of the screen. Stimuli were identical with those used in Task 4, but the sequence of presentation was different. The task design differed from that of Task 4 only in that participants were forced to decide whether the expression-mix stimulus was composed of more of the left or more of the right prototypical expression. Response keys were the left and right control keys on the regular computer keyboard, which were marked with colored tape.

#### Results and discussion

The average percentages of correct decisions are given in Table 2. This task was rather easy compared with Tasks 1–3. The distribution of the scores was, however, not strongly skewed to the right, but rather followed a normal distribution with most of the participants performing within the range of 0.70–0.85; therefore, this task can be used to measure individual differences in performance accuracy. An rmANOVA revealed that the expressed emotion affected recognition accuracy, [*F*_(5, 1340)_ = 172.94, *p* < 0.001, η^2^ = 0.33]. Similarly to Task 4, the rank order of emotion recognizability was not similar to Tasks 1 or 2. An rmANOVA with factor mixture ratio (levels corresponding to those from Task 4) showed a significant effect, [*F*_(4, 1072)_ = 101.95, *p* < 0.001, η^2^ = 0.21]. Discrimination rates were highest at mixture ratios of 95 and 85% and decreased with decreasing ratio of the prevailing emotion. Reliability estimates of the overall score were admissible (α = 0.63; ω = 0.64) but rather poor for the emotion-specific scores (see Table 2), probably due to several items with skewed distributions and thus poor psychometric quality. Generally, the psychometric properties of this task need improvement and further studies should address the question whether forced-choice expression assignment in emotion-morphs is indicating the same ability factor indicated by the other tasks (i.e., emotion identification and discrimination of prototypical expressions).

## Learning and recognition tasks of facial emotion expressions

The following five tasks arguably assess individual differences in memory-related abilities in the domain of facial expressions. All tasks consist of a learning phase for facial expressions and a subsequent retrieval phase that requires recognition or recall of previously learned expressions. The first three memory tasks include an intermediate task between learning and recall of at least three minutes, hence challenging long-term retention. In Task 9 and 10, learning is immediately followed by retrieval. These tasks should measure primary and secondary memory (PM and SM; Unsworth and Engle, 2007) of emotion expressions.

### Task 6: learning and recognition of emotion expressions of different intensity

With this forced-choice SM task we aimed to assess the ability to learn and recognize facial expressions of different intensity. Emotion category, emotion intensity, and learning-set size varied across trials, but face identity was constant within a block of expressions that the participant was asked to learn together. Manipulations of expression intensity within targets, but also between targets and distracters, were used to increase task difficulty. The recognition of expression intensity is also a challenge in everyday life; hence, the expression intensity manipulation is not restricted to psychometric rationales.

The combination of six emotional expressions with three intensity levels (low—the target emotion expression intensity was above 60%, medium—intensity above 80%, high—intensity above 95%) resulted in a matrix with 18 conceivable stimuli categories for a trial block. We expected hit-rates to decline with increasing ambiguity for less intense targets (e.g., see the effects of inter-item similarity on visual-memory for synthetic faces reported by Yotsumoto et al., 2007) and false alarm rates to grow for distracters of low intensity (e.g., see effects of target-distracter similarity in face recognition reported by Davies et al., 1979).

#### Procedure

We administered one practice block of trials and four experimental blocks—including four face identities (half were females) and 18 trials per block. Each block started by presenting a set of target faces of the same face identity but with different emotion expressions. To-be-learned stimuli were presented simultaneously in a line centered on the screen. Experimental blocks differed in the number of targets, expressed emotion, expression intensity, and presentation time. Presentation time ranged from 30 to 60 s depending on the number of targets within a block (two up to five stimuli). Facial expressions of six emotions were used as targets as well as distracters (happiness, surprise, anger, fear, sadness, and disgust).

Participants were instructed to remember the combination of both expression and intensity. During a delay phase of about three minutes, participants worked on a two-choice RT task (they had to decide whether two simultaneous presented number series are the same or different). Recall was structured as a pseudo-randomized sequence of 18 single images of targets or distracters. Targets were identical with the previously learned expressions in terms of emotional content and intensity, but different photographs of the same identities were used in order to reduce effects of simple image recognition. Distracters differed from the targets in both expression content and intensity. Participants were requested to provide a two-choice discrimination decision between learned and distracter expressions on the keyboard. After a response, the next stimulus was presented.

#### Results and discussion

The average performance accuracy over all trials and across trials of specific emotion categories is presented in Table 3. Emotion significantly affected recognition performance, [*F*_(5, 1340)_ = 120.63, *p* < 0.001, η^2^ = 0.24]. Pairwise comparison based on adjusted *p*-values for simultaneous inference using the Bonferroni-method showed that participants were better at recognizing happiness relative to all other emotions. Additionally, expressions of anger were significantly better retrieved than surprise, fear, or disgust expressions. There were no additional performance differences due to emotion content.

**Table 3:** **Descriptive statistics and reliability estimates of performance accuracy for all emotion memory tasks—across all trials and for single target emotions (if applicable)**.

**Condition**	**Accuracy *M* (*SD, SE*)**	**Alternative score *M* (*SD, SE*)**	**Alpha / Omega / # of Trials**
**TASK 6: LEARNING AND RECOGNITION OF EMOTION EXPRESSIONS OF DIFFERENT INTENSITY**			
Overall (range: 0.49–0.89)	0.76 (0.06, 0.00)	1.50 (0.63, 0.04)^**^	0.76/0.76/72
Happiness (1^*^)	0.89 (0.10, 0.01)	Overall accuracy and d'prime score: *r* = 0.72	0.50/0.53/10
Surprise (4)	0.73 (0.10, 0.01)		0.53/0.54/14
Fear (4)	0.73 (0.13, 0.01)		0.51/0.52/13
Sadness (3)	0.74 (0.11, 0.01)		0.49/0.50/11
Disgust (4)	0.73 (0.11, 0.01)		0.62/0.64/14
Anger (2)	0.76 (0.10, 0.01)		0.50/0.51/10
**TASK 7: LEARNING AND RECOGNITION OF EMOTIONAL EXPRESSIONS FROM DIFFERENT VIEWPOINTS**			
Overall (range: 0.46–0.91)	0.75 (0.08, 0.01)	1.46 (0.63, 0.04)^**^	0.75/0.75/56
Happiness (2)	0.79 (0.16, 0.01)	Overall accuracy and d'prime score: *r* = 0.94	0.34/0.38/08
Surprise (1^*^)	0.81 (0.15, 0.01)		0.45/0.46/08
Fear (5)	0.72 (0.14, 0.01)		0.27/0.28/10
Sadness (3)	0.77 (0.14, 0.01)		0.38/0.39/09
Disgust (4)	0.73 (0.14, 0.01)		0.44/0.45/12
Anger (6)	0.68 (0.14, 0.01)		0.26/0.30/09
**TASK 8: LEARNING AND RECOGNITION OF MIXED EMOTION EXPRESSIONS IN EXPRESSION MORPHS**			
Overall (range: 0.43–0.89)	0.69 (0.08, 0.00)	1.11 (0.55, 0.03)^**^	0.68/0.68/56
		Overall accuracy and d'prime score: *r* = 0.92	
**TASK 9: CUED EMOTIONAL EXPRESSIONS SPAN**			
Overall (range: 0.09–0.75)	0.45 (0.13, 0.01)	–	0.59/0.59/32
Happiness (1^*^)	0.54 (0.25, 0.02)	–	0.34/0.42/05
Surprise (2)	0.42 (0.23, 0.01)	–	0.33/0.34/06
Fear (4)	0.38 (0.22, 0.01)	–	0.16/0.23/05
Sadness (3)	0.39 (0.24, 0.01)	–	0.22/0.35/05
Disgust (1)	0.55 (0.22, 0.01)	–	0.29/0.31/06
Anger (3)	0.39 (0.22, 0.01)	–	0.16/0.18/05
**TASK 10: MEMORY FOR FACIAL EXPRESSIONS OF EMOTIONS**			
Overall (range: 0.07–0.78)	0.42 (0.13, 0.01)	–	0.58/0.60/27
Happiness (1^*^)	0.52 (0.26, 0.02)	–	0.20/0.37/04
Surprise (2)	0.46 (0.21, 0.01)	–	0.27/0.28/05
Fear (4)	0.42 (0.20, 0.01)	–	0.14/0.18/04
Sadness (6)	0.23 (0.21, 0.01)	–	0.20/0.22/03
Disgust (5)	0.38 (0.24, 0.01)	–	0.26/0.27/05
Anger (3)	0.44 (0.19, 0.01)	–	0.27/0.30/06

M, means, SE, standard errors, SD, standard deviations; Alpha (α) and Omega (ω) coefficients; # of Trials, the number of trials used for calculating the reliability coefficients;

* , the rank order of recognizability across emotions is indicated in the brackets;

** , d'prime score; the chance probability in case of Task 6, 7, and 8 is 0.50 and cannot be computed for tasks 9 and 10.

Intensity manipulation was partly successful: High intensity stimuli were recognized (*M*_95%_ = 0.86; *SD*_95%_ = 0.10) better than medium or low intensity stimuli (*M*_80%_ = 0.70; *SD*_80%_ = 0.08; *M*_60%_ = 0.74; *SD*_60%_ = 0.10); [*F*_(2, 536)_ = 236.24, *p* < 0.001, η^2^ = 0.35]. While performance on low intensity stimuli was slightly better than performance on medium intensity stimuli, we believe this effect reflects a Type 1 error and will not replicate in an independent sample. We recommend using high vs. low intensity (95 and 60% expressions) as difficulty manipulation in this task in future studies. Reliability estimates are provided in Table 3 and suggest good psychometric properties for the overall task. Reliabilities for emotion-specific trials are acceptable considering the low number of indicators and the heterogeneity of facial emotion expressions in general. In sum, we recommend using two levels of difficulty and the overall performance as indicators of expression recognition accuracy for this task.

### Task 7: learning and recognition of emotional expressions from different viewpoints

In this delayed recognition memory task, we displayed facial expressions with a frontal view as well as right and left three-quarter views. We aimed to assess long-term memory bindings between emotion expressions and face orientation. Thus, in order to achieve a correct response, participants needed to store both the emotion expressions and the viewpoints. This task is based on the premise that remembering content-context bindings is crucial in everyday socio-emotional interactions. An obvious hypothesis regarding this task is that emotion expressions are recognized more accurately from the frontal view than from the side, because more facial muscles are visible from the frontal view. On the other side, Matsumoto and Hwang (2011) reported that the presentation of emotional expressions in hemi-face profiles did not lower accuracy rates of recognition relative to frontal views. It is important to note that manipulation of the viewpoint is confounded with manipulating gaze direction in the present task. Adams and Kleck (2003) discuss effects of gaze direction on the processing of emotion expressions. A comparison of accuracy rates between frontal and three-quarter views is therefore interesting.

#### Procedure

This task includes one practice block and four experimental blocks with each including only one face identity and consisting of 12–16 recall-trials. During the initial learning phase, emotion expressions from different viewpoints were simultaneously presented. The memory set size varied across blocks from four to seven target stimuli. Targets differed according to the six basic emotions and the three facial perspectives (frontal, left, and right profile views). Presentation time changed depending on the number of stimuli presented during the learning phase and ranged between 30 and 55 s. Participants were explicitly instructed to memorize the association between expressed emotion and perspective. This was followed by a verbal two-choice RT distracter task for about one minute where participants decided whether a presented word contained the letter “A.” During the recall phase, novel images, which showed the emotion expression from the same perspective as in the learning phase, served as the targets. These images were shown in a pseudo-randomized sequence intermixed with distracters, which differed from the targets in expression, perspective, or both. Participants were asked to decide whether or not a given image had been shown during the learning phase by pressing one of two buttons on the keyboard. After the participants chose their response, the next trial started.

#### Results and discussion

Table 3 displays the performance accuracy for this task. The average scores suggest adequate levels of task difficulty—well above guessing probability and below ceiling. Reliability estimates for the overall task reveal good psychometric quality (α = 0.75; ω = 0.75). Reliability estimates for the emotion specific trials were considerably lower; these estimates might be raised by increasing the number of stimuli per emotion category. An rmANOVA suggested rather small but significant performance differences between emotion categories, [*F*_(5, 1340)_ = 38.06, *p* < 0.001, η^2^ = 0.09]. Pairwise comparisons showed that expressions of happiness and surprise were recognized the best and anger and fear were recognized the worst. Viewpoint effects were as expected and contradict the results from Matsumoto and Hwang (2011). Expressions were recognized significantly better if the expression was learned with frontal rather than a three quarter view: [*F*_(1, 268)_ = 13.60, *p* < 0.001], however the mean difference between these two perspectives was low (*M*_diff_ = 0.03; η^2^ = 0.02). We recommend using face orientation as a difficulty manipulation and the overall performance across trials as indicator of expression recognition.

### Task 8: learning and recognition of mixed emotion expressions in expression morphs

With this task, we intended to assess recognition performance of mixed—rather than prototypical—facial expressions. It was not our aim to test theories that postulate combinations of emotions that result in complex affect expressions, such as contempt, which is proposed as a mixture of anger and disgust or disappointment, which is proposed as a combination of surprise and sadness (cf. (Plutchik, 2001)). Instead, we aimed to use compound emotion expressions to assess the ability to recognize less prototypical, and to some extent more real-life, expressions. Furthermore, these expressions are not as easy to label as the basic emotion expressions parenting the mixed expressions. Therefore, for the mixed emotions of the present task we expect a smaller contribution of verbal encoding to task performance, as has been reported for face recognition memory for basic emotions (Nakabayashi and Burton, 2008).

#### Procedure

We used nine different combinations of six basic emotions (Plutchik, 2001): Fear/disgust, fear/sadness, fear/happiness, fear/surprise, anger/disgust, anger/happiness, sadness/surprise, sadness/disgust, and happiness/surprise. Within each block of trials, the images used for morphing mixed expressions were from a single identity. Across blocks, sex of the identities was balanced. There were four experimental blocks preceded by a practice block. The number of stimuli to be learned ranged from two targets in Block 1 to five targets in Block 4. The presentation time of the targets during the learning period changed depending on the number of targets displayed, ranging from 30 to 60 s. Across blocks, 11 targets showed morphed mixture ratios of 50:50 and five targets showed one dominant expression. During the learning phase, stimuli were presented simultaneously on the screen. During a delay period of approximately three minutes, participants answered a subset of questions from the Trait Meta Mood Scale (Salovey et al., 1995). At retrieval, participants saw a pseudo-randomized sequence of images displaying mixed expressions. Half of the trials were learned images. The other trials differed from the learned targets in the expression mixture, in the mixture ratio, or both. Participants were asked to decide whether or not a given image had been shown during the learning phase by pressing one of two buttons on the keyboard. There were 56 recall trials in this task. The same scoring procedures were used as in Task 7.

#### Results and discussion

The average performance over all trials (see Table 3) was well above chance. Different scoring procedures hardly affected the rank order of individuals within the sample; the proportion correct scores were highly correlated with the d'prime scores (0.92; *p* < 0.001). Reliability estimates suggest good psychometric quality. Further studies are needed to investigate whether learning and recognizing emotion-morphs are tapping the same ability factor as learning and recognizing prototypical expressions of emotion. Because expectations on mean differences at recognizing expression morphs are difficult to derive from a theoretical point of view, we only consider the psychometric quality of the overall score for this task.

### Task 9: cued emotional expressions span

Memory span paradigms are frequently used measures of primary memory. The present task was designed as a serial cued memory task for emotion expressions of different intensity. Because recognition was required in the serial order of the stimuli displayed at learning, the sequence of presentation served as a temporal-context for memorizing facial expressions. We used FaceReader (see above) to score intensity levels of the stimuli chosen for this task.

#### Procedure

Based on the FaceReader scores, we categorized the facial stimuli into low intensity (60%), medium intensity (70%), and high intensity (80%) groups. We used three male and four female identities throughout the task, with one identity per block. The task began with a practice block followed by seven experimental blocks of trials. Each block started with a sequence of facial expressions (happiness, surprise, fear, sadness, disgust, and anger), presented one at a time, and was followed immediately by the retrieval phase. The sequence of targets at retrieval was the same as the memorized sequence. Participants were suggested to use the serial position as memory cue. Number of trials within a sequence varied between three and six. Most of the targets (25 of 33 images) and distracters (37 of 54 images) displayed high intensity prototypical expressions.

During the learning phase stimulus presentation was fixed to 500 ms, followed by a blank inter-stimulus interval of another 500 ms. At retrieval, the learned target-expression was shown simultaneously with three distractors in a 2 × 2 matrix. The position of the target in this matrix varied across trials. Distracters within a trial differed from the target in its emotional expression, intensity, or both. Participants indicated the learned expression via mouse click on the target image.

#### Results and discussion

Table 3 provides performance and reliability estimates. Average performance ranged between 0.38 and 0.55, was clearly above chance level, and with no ceiling effect. Reliability estimates for the entire task are acceptable; reliability estimates for the emotion-specific trials were low; increasing the number of trials could improve reliabilities for the emotion-specific trials. An rmANOVA suggested significant but small performance differences across the emotion categories, [*F*_(5, 1340)_ = 36.98, *p* < 0.001, η^2^ = 0.09]; pairwise comparisons revealed participants were better at retrieving happiness and disgust expressions compared with all other expressions; no other differences were statistically significant. We therefore recommend the overall percentage correct score as a psychometrically suitable measure of individual differences of primary memory for facial expressions.

### Task 10: the game memory with emotion expressions

This task is akin to the well-known game “Memory.” Several pairs of emotion expressions, congruent in emotional expression and intensity, were presented simultaneously for a short time. The task was to quickly detect the particular pairs and to memorize them in conjunction with their spatial arrangement on the screen. Successful detection of the pairs requires perceptual abilities. During retrieval, one expression was automatically disclosed and participants had to indicate the location of the corresponding expression. Future work might decompose perceptual and mnestic demands of this task in a regression analysis.

#### Procedure

**Figure 4:**
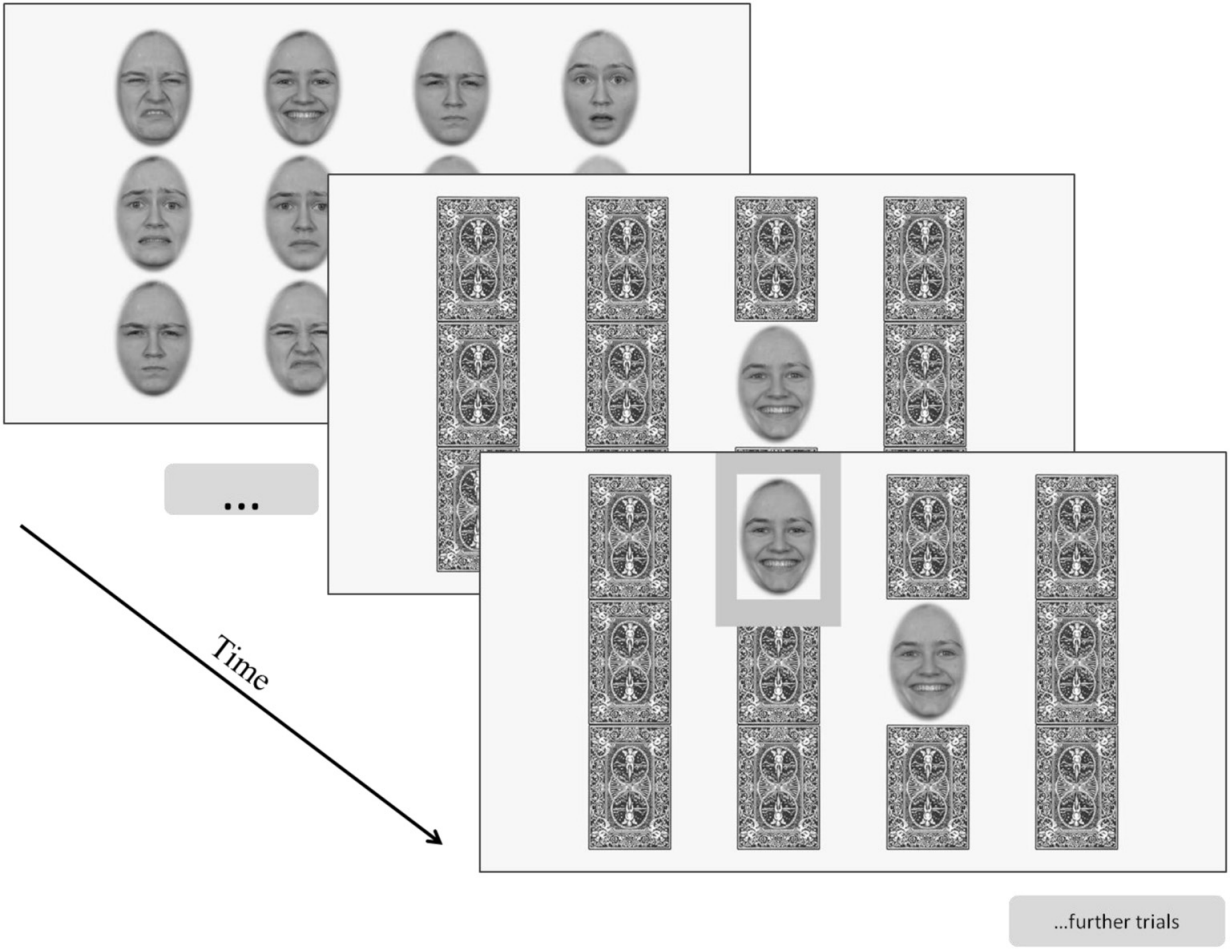
**Schematic representation of a trail block from Task 10 (Memory for facial expression of emotion)**.

At the beginning of a trial block several expressions initially covered with a card deck appeared as a matrix on the screen. During the learning phase, all expressions were automatically disclosed and participants were asked to detect expression pairs and to memorize their location. Then, after several seconds, the learning phase was stopped by the program, and again the cards were displayed on the screen. Next, one image was automatically disclosed and participants indicated the location of the corresponding expression with a mouse click. After the participant's response, the clicked image was revealed and feedback was given by encircling the image in green (correct) or red (incorrect). Two seconds after the participant responded, the two images were again masked with the cards, and the next trial started by program flipping over another card to reveal a new image. Figure 4 provides a schematic representation of the trial sequence within an experimental block.

Following the practice block, there were four experimental blocks of trials. Expression matrices included three (one block), six (one block), and nine (two blocks) pairs of expressions that were distributed pseudo-randomized across the lines and columns. Presentation time for learning depended on the memory set size: 20, 40, and 60 s for three, six, and nine expression pairs, respectively. Within each block each image pair was used only once, resulting in 27 responses, representing the total number of trials for this task.

#### Results and discussion

The average proportion of correctly identified emotion pairs and reliability estimates, are summarized in Table 3. Similar to Task 9, guessing probability is much lower than 0.50 in this task; therefore the overall accuracy of 0.40 is acceptable. Reliability is also good. Due to the low number of trials within one emotion category, these reliabilities are rather poor, but could be increased by including additional trials. There was a small but significant effect of emotion category on performance accuracy, [*F*_(5, 1340)_ = 68.74, *p* < 0.001, η^2^ = 0.15], as indicated by an rmANOVA. Pairs of happiness, surprise, anger, and fear expressions were remembered the best and sadness was remembered the worst. In the current version, we recommend the overall score as a psychometrically suitable performance indicator of memory for emotional expressions.

## Speed tasks of perceiving and identifying facial emotion expressions

We also developed speed indicators of emotion perception and emotion recognition ability following the same rationale as described by Herzmann et al. (2008) and Wilhelm et al. (2010). Tasks that are so easy that the measured accuracy levels are at ceiling allow us to gather individual differences in performance speed. Therefore, for the following tasks we used stimuli with high intensity prototypical expressions for which we expected recognition accuracy rates to be at or close to ceiling (above 0.80). Like the accuracy tasks described above, the speed tasks were intended to measure either emotion perception (three tasks) or emotion recognition (three tasks). Below we describe the six speed tasks and report results on their psychometric properties.

### Task 11: emotion perception from different viewpoints

Recognizing expressions from different viewpoints is a crucial socio-emotional competence relevant for everyday interaction. Here, we aimed to assess the speed of perceiving emotion expressions from different viewpoints by using a discrimination task with same-different choices.

#### Procedure

Two same-sex images with different facial identities were presented next to each other. One face was shown with a frontal view and the other in three-quarter view. Both displayed one of the six prototypical emotion expressions. Participants were asked to decide as fast and accurately as possible whether the two persons showed the same or different emotion expressions by pressing one of two marked keys on the keyboard.

There was no time limit on the presentation. Participants' response started the next trial, after the presentation of a 1.3 s blank interval. Trials were pseudo-randomized in sequence and were balanced for expression match vs. mismatch, side of presentation, position of the frontal and three-quarter view picture, as well as for the face identity sex. To ensure high accuracy rates, confusable expressions according to the hexagon model (Sprengelmeyer et al., 1996) were never presented together in mismatch trials. There was a practice block of six trials with feedback. Experimental trials started when the participant achieved a 70% success rate in the practice trials. There were 31 experimental trials. Each of the six basic emotions occurred in match and mismatch trials.

#### Results and discussion

Average accuracies and RTs, along with average inverted latency (see general description of scoring procedures above) are presented in Table 4. As required for speed tasks, accuracy rates were at ceiling. RTs and inverted latencies showed that participants needed about two seconds on average to correctly match the two facial expressions presented in the frontal vs. three quarter view. An rmANOVA of inverted RTs revealed differences in the expression matching speed across emotion categories, [*F*_(5, 1340)_ = 263.84, *p* < 0.001, η^2^ = 0.22]. Bonferroni-adjusted pairwise comparisons indicate the strongest difference in performance between matching emotion expressions occurred between happiness compared to all other emotions. Other statistically significant, but small effects indicated that performance matching surprise, fear, and anger expressions was faster than performance matching sadness and disgust. Reliability estimates are excellent for the overall score and acceptable for the emotion specific trials. However, happiness, surprise, and fear expressions were less frequently used in this task. Reliabilities of emotion-specific scores could be increased by using more trials in future applications.

**Table 4:** **Descriptive statistics and reliability estimates of performance speed for all speed measures of emotion perception—across all trials and for single target emotions**.

**Condition**	**Accuracy *M* (*SD, SE*)**	**Reaction time; 1000/reaction time; *M* (*SD, SE*)**	**Alpha / Omega / # of Trials**
**TASK 11: EMOTION PERCEPTION FROM DIFFERENT VIEWPOINTS**			
Overall	0.90 (0.06, 0.00)	2181 (686, 41) 0.59 (0.15, 0.01)	0.95/0.96/31
Happiness	0.98 (0.09, 0.01)	1325 (549, 34) 0.86 (0.23, 0.01)	0.66/0.66/02
Surprise	0.94 (0.17, 0.01)	1966 (818, 53) 0.62 (0.20, 0.01)	0.51/0.51/02
Fear	0.95 (0.11, 0.01)	2018 (733, 49) 0.62 (0.17, 0.01)	0.46/0.47/04
Sadness	0.90 (0.11, 0.01)	2426 (841, 21) 0.58 (0.18, 0.01)	0.88/0.88/09
Disgust	0.85 (0.13, 0.01)	2115 (673, 76) 0.58 (0.15, 0.01)	0.78/0.79/07
Anger	0.96 (0.08, 0.01)	2033 (638, 45) 0.62 (0.16, 0.01)	0.82/0.83/07
**TASK 12: EMOTIONAL ODD-MAN-OUT**			
Overall	0.96 (0.05, 0.00)	2266 (761, 46) 0.56 (0.15, 0.01)	0.96/0.96/30
Happiness	0.98 (0.06, 0.00)	1844 (654, 42) 0.65 (0.18, 0.01)	0.72/0.73/05
Surprise	0.95 (0.13, 0.01)	2224 (936, 71) 0.54 (0.16, 0.01)	0.73/0.73/05
Fear	0.94 (0.12, 0.01)	2672 (983, 74) 0.49 (0.16, 0.01)	0.67/0.67/05
Sadness	0.95 (0.10, 0.01)	2453 (916, 62) 0.53 (0.18, 0.01)	0.75/0.75/05
Disgust	0.98 (0.06, 0.00)	2252 (901, 50) 0.57 (0.18, 0.01)	0.70/0.71/05
Anger	0.97 (0.08, 0.00)	2301 (940, 61) 0.57 (0.18, 0.01)	0.72/0.73/05
**TASK 13: IDENTIFICATION SPEED OF EMOTIONAL EXPRESSIONS**			
Overall	0.93 (0.05, 0.00)	3043 (774, 47) 0.42 (0.09, 0.01)	0.96/0.96/48
Happiness	0.98 (0.05, 0.00)	1893 (548, 35) 0.63 (0.16, 0.01)	0.78/0.79/08
Surprise	0.99 (0.04, 0.00)	2921 (832, 53) 0.43 (0.11, 0.01)	0.77/0.78/08
Fear	0.85 (0.16, 0.01)	4078 (955, 96) 0.30 (0.08, 0.00)	0.77/0.77/08
Sadness	0.90 (0.11, 0.01)	3462 (952, 90) 0.35 (0.10, 0.01)	0.78/0.78/08
Disgust	0.95 (0.08, 0.00)	2794 (791, 60) 0.42 (0.10, 0.01)	0.78/0.78/08
Anger	0.90 (0.12, 0.01)	3281 (948, 81) 0.36 (0.10, 0.01)	0.81/0.81/08

M, means, SE, standard errors, SD, standard deviations; Alpha (α) and Omega (ω) coefficients calculated for speed measures (1000/reaction time); # of Trials, the number of trials used for calculating reliability coefficients.

### Task 12: emotional odd-man-out

This task is a revision of the classic Odd-Man-Out task (Frearson and Eysenck, 1986), where several items are shown simultaneously of which one—the odd-man-out—differs from the others. Participants' task is to indicate the location of the odd-man-out. The emotion-expression version of the task—as implemented by Herzmann et al. (2008) and in the present study—requires distinguishing between different facial expressions of emotion presented within a trial in order to detect the “odd” emotional expression.

#### Procedure

Three faces of different identities (but of the same sex), each displaying an emotion expression, were presented simultaneously in a row on the screen. The face in the center displayed the reference emotion from which either the left or right face differed in expression, whereas the remaining third face displayed the same emotion. Participants had to locate the divergent stimulus (odd-man-out) by pressing a key on the corresponding side. The next trial started after a 1.3-s blank interval. Again, we avoided combining highly confusable expressions of emotions in the same trial to ensure high accuracy rates (Sprengelmeyer et al., 1996). Five practice trials with feedback and 30 experimental trials were administered in pseudo-randomized order. Each emotion occurred as both a target and as a distracter.

#### Results and discussion

Table 4 displays relevant results for this task. Throughout, accuracy rates were very high for all performance indicators, demonstrating the task to be a measure of performance speed. On average, participants needed about 2 s to detect the odd-man-out. There were statistically significant, but small performance differences depending on the emotion category, [*F*_(5, 1340)_ = 109.43, *p* < 0.001, η^2^ = 0.08]. Differences mainly occurred between happiness and all other expressions. In spite of the small number of trials per emotion category (5), reliability estimates of the overall score based on inverted latencies are excellent and good for all emotion specific scores. We conclude that the overall task and emotion specific trial scores have good psychometric quality.

### Task 13: identification speed of emotional expressions

The purpose of this task is to measure the speed of the visual search process (see Task 3) involved in identifying an expression belonging to an indicated expression category. Here, an emotion label, a targeted emotional expression, and three mismatching alternative expressions, were presented simultaneously on the screen. The number of distracters was low in order to minimize task difficulty. Successful performance on this task requires a correct link of the emotion label and the facially expressed emotion and an accurate categorization of the expression to the appropriate semantic category.

#### Procedure

The name of one of the six basic emotions was printed in the center of the screen. The emotion label was surrounded in horizontal and vertical directions by four different face identities of the same sex all displaying different emotional expressions. Participants were asked to respond with their choice by using the arrow-keys on the number block of a regular keyboard. There were two practice trials at the beginning. Then, each of the six emotions was used eight times as a target in a pseudorandom sequence of 48 experimental trials. There were no time limits for the response, but participants were instructed to be as fast and accurate as possible. The ISI was 1300 ms.

#### Results and discussion

Average performance, as reflected by the three relevant scores for speed indicators, are depicted in Table 4. Accuracy rates were at ceiling. An rmANOVA of inverted latencies showed strong differences in performance speed for the different emotional expressions, [*F*_(5, 1340)_ = 839.02, *p* < 0.001, η^2^ = 0.48]. Expressions of happiness and surprise were detected the fastest, followed by disgust and anger, and finally sadness and fear. Reliability estimates were excellent for the overall score and good for emotion specific performance scores. All results substantiate that the scores derived from Task 12 reflect the intended difficulty for speed tasks and have good psychometric properties.

## Speed tasks of learning and recognizing facial emotion expressions

### Task 14: *1*-back recognition speed of emotional expressions

In the *n*-back paradigm, a series of different pictures is presented; the task is to judge whether a given picture has been presented *n* pictures before. It has been traditionally used to measure working memory (e.g., Cohen et al., 1997). The *1*-back condition requires only minimal effort on storage and processing in working memory. Therefore, with the *1*-back task using emotion expressions we aimed to assess recognition speed of emotional expressions from working memory and expected accuracy levels to be at ceiling.

#### Procedure

We administered a *1*-back task with one practice block and four experimental blocks of trials. Each experimental block consisted of a sequence of 24 different images originating from the same identity displaying all six facial emotional expressions. Participants were instructed to judge whether the emotional expression of each image was the same as the expression presented in the previous trial. The two-choice response was given with a left or right key (for mismatches and matches, respectively) on a standard keyboard. The next trial started after the participant provided their response, with a fixation cross presented on a blank screen for 200 ms in between trials. Response time was not limited by the experiment. Practice trials with feedback needed to be completed with at least 80% accuracy in order to continue with the experimental blocks. All basic emotion expressions were presented as targets in at least one experimental block. Target and distracters were presented at a ratio of 1:4 and there were 24 target trials in total.

#### Results and discussion

Table 5 summarizes the average accuracies, RTs, and inverted latencies. As expected, accuracies were at ceiling. Participants were on average able to correctly respond to more than one trial per second. There were very small (but statistically significant) differences between emotion categories, as suggested by an rmANOVA, [*F*_(5, 1340)_ = 29.99, *p* < 0.001, η^2^ = 0.04]. Reliability estimates were excellent for the overall task and acceptable for emotion specific latency scores given the low number of trials for an emotion category. These results suggest that Task 14 is a psychometrically sound measure of emotion recognition speed from faces.

**Table 5:** **Mean accuracy, reaction times (in ms) and reliability estimates of performance speed for all speed measures of emotion memory—across all trials and for single target emotions (if applicable)**.

**Condition**	**Accuracy *M* (*SD, SE*)**	**Reaction time; 1000/reaction time; *M* (*SD, SE*)**	**Alpha / Omega / # of Trials**
**TASK 14: *1*-back RECOGNITION SPEED OF EMOTIONAL EXPRESSIONS**			
Overall	0.94 (0.05, 0.00)	880 (193, 11) 1.17 (0.25, 0.02)	0.91/0.91/24
Happiness	0.88 (0.18, 0.01)	923 (337, 20) 1.26 (0.26, 0.02)	0.65/0.65/04
Surprise	0.85 (0.19, 0.01)	867 (515, 31) 1.34 (0.27, 0.02)	0.64/0.64/04
Fear	0.88 (0.18, 0.01)	941 (269, 16) 1.21 (0.23, 0.01)	0.58/0.58/04
Sadness	0.92 (0.15, 0.01)	807 (186, 11) 1.36 (0.25, 0.02)	0.62/0.62/04
Disgust	0.93 (0.16, 0.01)	833 (228, 14) 1.34 (0.25, 0.02)	0.55/0.58/04
Anger	0.93 (0.15, 0.01)	876 (285, 17) 1.31 (0.23, 0.01)	0.64/0.65/04
**TASK 15: DELAYED NON-MATCHING TO SAMPLE WITH EMOTIONAL EXPRESSIONS**			
Overall	0.90 (0.08, 0.00)	1555 (412, 25) 0.80 (0.20, 0.01)	0.95/0.96/36
Happiness	0.95 (0.11, 0.01)	1159 (392, 23) 1.00 (0.27, 0.02)	0.87/0.87/06
Surprise	0.97 (0.09, 0.01)	1385 (436, 26) 0.85 (0.24, 0.01)	0.84/0.84/06
Fear	0.93 (0.13, 0.01)	1474 (491, 29) 0.82 (0.24, 0.01)	0.81/0.82/06
Sadness	0.75 (0.23, 0.01)	2232 (907, 55) 0.59 (0.21, 0.01)	0.78/0.79/06
Disgust	0.85 (0.17, 0.01)	1930 (671, 40) 0.64 (0.20, 0.01)	0.77/0.77/06
Anger	0.91 (0.14, 0.01)	1589 (492, 30) 0.73 (0.21, 0.01)	0.76/0.76/06
**TASK 16: RECOGNITION SPEED OF MORPHED EMOTIONAL EXPRESSIONS**			
Overall	0.89 (0.09, 0.01)	1068 (228, 13) 1.25 (0.20, 0.01)	0.92/0.92/36
Happiness—surprise	0.89 (0.17, 0.01)	870 (269, 16) 1.42 (0.25, 0.02)	0.63/0.64/07
Happiness—anger	0.89 (0.19, 0.01)	948 (307, 18) 1.27 (0.25, 0.02)	0.64/0.65/05
Fear—surprise	0.90 (0.12, 0.01)	958 (290, 17) 1.33 (0.25, 0.02)	0.71/0.72/07
Fear—sadness	0.89 (0.17, 0.01)	932 (270, 16) 1.31 (0.27, 0.02)	0.67/0.68/05
Sadness—disgust	0.84 (0.21, 0.01)	1488 (685, 41) 0.95 (0.25, 0.01)	0.60/0.62/05
Disgust—anger	0.93 (0.11, 0.01)	1264 (380, 23) 1.12 (0.22, 0.01)	0.64/0.64/07

M, means, SE, standard errors, SD, standard deviations; Alpha (α) and Omega (ω) coefficients calculated for speed measures (1000/reaction time); # of Trials, the number of trials used for calculating the reliability coefficients.

### Task 15: delayed non-matching to sample with emotional expressions

The present task was inspired by the Delayed Non-Matching paradigm implemented for face identity recognition by Herzmann et al. (2008) and was modified here in order to assess emotion expression recognition. This task requires the participant to store and maintain a memory of each emotion expression; the images are presented during the learning phase for a short period of time and during the experimental trials the images have to be recollected from the visual primary memory and compared with a novel facial expression. Because the task requires a short maintenance time for a single item in the absence of interfering stimuli, we expect the task to show accuracy rates at ceiling and to measure short-term recognition speed.

#### Procedure

A facial expression of happiness, surprise, fear, sadness, disgust, or anger was presented for 1 second. Following a delay of 4 s (500 ms mask; 3500 ms blank screen) the same emotion expression was presented together with a different facial expression. Depending on where the new (distracter) expression was presented, participants had to press a left or right response-key on a standard keyboard in order to indicate the distractor facial expression. In each trial we used three different identities of the same sex. During the 36 experimental trials, expressions belonging to each emotion category had to be encoded six times. There were three practice trials.

#### Results and discussion

Results are summarized in Table 5. Average accuracy across participants suggests ceiling effects for recognizing emotion expressions, with the exception of sadness where recognition rates were rather low. Speed of sadness recognition should be carefully interpreted because it relies on just a few latencies associated with correct responses for many of the participants. Overall, participants correctly recognized less than one item per second and exactly one item per second in the case of happy faces. There were medium-sized differences in performance speed across emotion categories, [*F*_(5, 1340)_ = 296.36, *p* < 0.001, η^2^ = 0.26], and pairwise comparisons suggested statistically significant differences in the recognition speed for all emotions except for fear and surprise. The rank order of recognition speed of the six emotions followed a pattern comparable to the pattern identified for the emotion perception accuracy tasks reported earlier. Divergent stimuli compared to happiness, surprise (and fear) as target expressions were identified the quickest, followed by anger, disgust, and finally sadness. Reliability estimates are again excellent for the overall score and very good for emotion specific trials, suggesting good psychometric quality.

### Task 16: recognition speed of morphed emotional expressions

This task is an implementation of a frequently used paradigm for measuring recognition memory (e.g., Warrington, 1984) and has also been used by Herzmann et al. (2008) for face identity recognition. The present task is derived from the face identity task and applied for emotion processing. We used morphed emotion expressions resulting in combinations of happiness, surprise, fear, sadness, disgust, or anger that were designed to appear as naturalistic as possible. Because the stimuli do not display prototypical expressions, the emotional expressions are difficult to memorize purely on the basis of semantic encoding strategies. The goal of this task was to measure visual encoding and recognition of facial expressions. In order to keep the memory demand low and to design the task to be a proper measure of speed, single expressions were presented for a relatively long period during the learning phase.

#### Procedure

This task consisted of one practice block and six experimental blocks. We kept stimulus identity constant within blocks. A block started with a 4-s learning phase, followed by a short delay during which participants were asked to answer two questions from a scale measuring extraversion, and finally the recognition phase. The extraversion items were included as an intermediate task in order to introduce a memory consolidation phase. There was one morphed expression to memorize per block during the 4-s learning time. The morphs were generated as a blend of two equally weighted, easily confusable emotional expressions according to their proximity on the emotion hexagon (Sprengelmeyer et al., 1996). During retrieval, the identically morphed expression was presented three times within a pseudo-randomized sequence with three different distracters. This resulted in six (targets and distracters) × six (blocks of trials) = 36 trials in total. All stimuli were presented in isolation during the recognition phase, each requiring a response. Participants indicated via a key press whether or not the presented stimulus was included in the learning phase at the beginning of the block. There were no restrictions on response time.

#### Results and discussion

Average performance, in terms of accuracy and the two different speed scores, are presented in Table 5. As expected, accuracy rates were at ceiling and participants were able to respond correctly to somewhat more than one trial per second on average. Specific morphs of different mixtures of emotion categories modulated performance differences in recognition speed; effects were of medium size, [*F*_(5, 1340)_ = 306.50, *p* < 0.001, η^2^ = 0.28]. Reliabilities were excellent for the overall score of inverted latencies and in the acceptable range for emotion-specific trials. The indicators derived from this task are therefore suitable measures of the speed of emotion recognition from faces.

## General discussion

We begin this discussion by providing a summary and evaluation of key findings and continue with methodological considerations regarding the overarching goal of this paper. We conclude with delineating some prospective research questions.

### Summary of key findings

We designed and assessed 16 tasks developed to measure individual differences in the ability to perceive or recognize facial emotion expressions. Each task explicitly measures these abilities by provoking maximum effort in participants and each item in each task has a veridical response. Performance is assessed by focusing on either the accuracy or speed of response. Competing approaches to scoring the measures were considered and compared for several tasks. The final set of suggested scoring procedures are psychometrically sound, simple, adequately distributed for future multivariate analysis, and exhaust the information collected. Therefore, all tasks can be considered to be measures of abilities. For each of the tasks we presented emotion-specific (where applicable) and overall scores concerning mean performance, individual differences in performance, and precision. Additionally, coefficients of internal consistency and factor saturation were presented for each task—including emotion-specific results when possible.

Taken together, the 16 tasks worked well: They were neither too easy nor too hard for the participants, and internal consistency and factor saturation were satisfactory. With respect to mean performance across all emotion domains and tasks there was an advantage for happy faces in comparison to all other facial expressions. This finding parallels several previous reports of within- and across-subject studies on facial expression recognition (e.g., Russell, 1994; Elfenbein and Ambady, 2002b,c; Jack et al., 2009; Recio et al., 2013). With respect to results concerning the covariance structure it might be argued that some of the emotion-specific results are not promising enough because some of the psychometric results are still in the lower range of desirable magnitudes. However, the tasks presented here ought not to be considered as stand-alone measures. Instead, preferably a compilation of these tasks should be jointly used to measure important facets of emotion-related interpersonal abilities. Methodologically, the tasks presented here would thus serve like the items of a conventional test as indicators below presupposed latent factors. Additionally, some of the unsatisfactory psychometric coefficients are likely to improve if test length is increased. Depending on available resources in a given study or application context and in line with the measurement intention tasks for one or more ability domains can be sampled from the present collection. We recommend sampling three or more tasks per ability domain. The duration estimates provided in Table 1 facilitate compilation of such task batteries in line with pragmatic needs of a given study or application context.

### Assessment of the overarching goal to develop a battery of indicators for perception and recognition of facially expressed emotions

In this paper, we presented a variety of tasks for the purpose of capturing individual differences in emotion perception and emotion recognition. The strategy in developing the present set of tasks was to sample measures established in experimental psychology and to adapt them for psychometric purposes. It is important to note that the predominant conceptual model in individual differences psychology presupposes effect indicators of common constructs. In these models, individual differences in indicators are caused by individual differences in at least one latent variable. Specific indicators in such models can be conceived as being sampled from a domain or range of tasks. Research relying on single indicators sample just one task from this domain and are therefore analogous to single case studies sampling just a single person. The virtue of sampling more than a single task is that further analysis of a variety of such measures allows abstracting not only from measurement error but also from task specificity.

In elaboration of this sampling concept we defined the domain from which we were sampling tasks a priori. Although general principles of sampling tasks from a domain have been specified, implicitly by Campbell and Fiske (1959) and Cattell (1961) and more explicitly by Little et al. (1999), the precise definition of “domain” is still opaque. In the present context, we applied a first distinction, which is well established in research on individual differences in cognitive abilities, namely between speed and accuracy tasks. A second distinction is based on the cognitive demand (perception vs. recognition) of an indicator. A speed task is defined as being so simple that members of the application population complete all tasks correctly if given unlimited time. An accuracy task is defined as being so hard that a substantial proportion of the application population cannot complete it correctly even if given unlimited time. We expect that once the guidelines and criteria suggested for tasks in the introduction are met and the following classifications of demands are applied—(a) primarily assessing emotion perception or emotion recognition and (b) provoking behavior that can be analyzed by focusing on either speed or accuracy, no further substantial determinants of individual differences can be established. Therefore, we anticipate that the expected diversity (Little et al., 1999) in the four domains distinguished here is low. This statement might seem very bold but we need to derive and test such general statements in order to avoid mixing up highly specific indicators with very general constructs. Obviously, this statement about task sampling applies to measures already developed (some of which were discussed in the introduction) and to measures still to be developed. A broad selection of tasks can be seen as a prerequisite to firmly establish the structure of individual differences in a domain under investigation. This is vividly visible in review work on cognitive abilities (Carroll, 1993).

It is not yet sufficiently clear how the arguments concerning task sampling apply to prior work on individual differences in socio-emotional abilities. Contemporary theories of socio-emotional abilities (e.g., Zeidner et al., 2008) clearly place the abilities investigated here in the realm of emotional intelligence. For example, the currently most prominent theory of emotional intelligence—the four-branch model by Mayer et al. (1999)—includes emotion perception as a key factor. It is important to note that many of the prevalent measures of emotional intelligence, which rely on self-report of typical behavior, do not meet standards that should be applied to cognitive ability measures (Wilhelm, 2005). Assessment tools of emotional intelligence, which utilize maximum effort performance in ability measures, meet some but not all of these criteria (Davies et al., 1998; Roberts et al., 2001). Arguably, these standards are met by the tasks discussed in the theoretical section and newly presented here. A convergent validation of the tasks presented here with popular measures of emotional intelligence—such as emotion perception measures in the MSCEIT (Mayer et al., 1999; MacCann and Roberts, 2008)—is therefore not as decisive as it usually is.

## Future research directions

This paper presents a compilation of assessment tools. Their purpose is to allow a psychologically based and psychometrically sound measurement of highly important receptive socio-emotional abilities, that is, the perception and recognition of facial expressions of emotions. We think that the present compilation has a variety of advantages over available measures and we stressed these advantages at several places in this paper. The goal was to provide a sufficiently detailed description of the experimental procedures for each task, to provide difficulty and reliability estimates for tasks and emotion specific subscales created within tasks. In further research we will consider the factorial structure across tasks and investigate competing measurement models of emotion perception and recognition—applying theoretically important distinctions between speed vs. accuracy and perception vs. recognition. An essential and indispensable step for such further research is the close inspection of the psychometric quality of each task.

The application populations of the present tasks are older adolescents and adults and task difficulty was shown to be somewhere between adequate and optimal for younger adults included in the present sample. With some adaptations the tasks can be applied to other populations too. The goal of our research is met best, when these tools (and adaptations or variants of them) are frequently used in many different research fields. We will briefly present some research directions we currently pursue in order to illustrate potential uses of our battery.

One question we are currently investigating is the distinction between the perception and recognition of unfamiliar neutral faces (Herzmann et al., 2008; Wilhelm et al., 2010) and the perception and recognition of unfamiliar emotional faces. Investigating such questions with psychometric methods is important in order to provide evidence that facial emotion reception is a determinant of specific individual differences. In elaboration of this research branch we also study individual differences in posing emotional expressions (Olderbak et al., 2013).

Obviously, the measures presented in this paper are also a promising approach when studying group differences—for example when studying differences between psychopathic and unimpaired participants (Marsh and Blair, 2008). Establishing deficient socio-emotional (interpersonal) abilities as key components of mental disorders hinges upon a solid measurement in many cases. We hope that the present contribution helps to provide such measurements.

Finally, we want to mention two important restrictions of the face stimuli used in the present tasks. First, the stimuli are exclusively portraits of white young adult middle-Europeans. The “Own-Race bias” (Meissner and Brigham, 2001) is a well-established effect in identity processing and it also applies to tasks capturing emotion perception (Elfenbein and Ambady, 2002a). It would therefore be adequate to create additional stimuli showing subjects of other ethnicity. Second, faces were presented without any context information, which—in most cases—enhances the reliability of their expressed (emotional) meanings (cf. Walla and Panksepp, 2013). Obviously, the tasks presented here could be used with stimulus sets varying in origin, ethnicity, color, age etc., and most of them could be extended to stimuli including varying contexts. Software code and more detail concerning the experimental setup and task design are available from the authors upon request.

## Author note

We thank Astrid Kiy, Thomas Lüttke, Janina Künecke, Guillermo Recio, Carolyn Nelles, Ananda Ahrens, Anastasia Janzen, Rosi Molitor, Iman Akra, and Anita Gazdag for their help with preparing the study and acquiring the data. This research was supported by a grant from the Deutsche Forschungsgemeinschaft (Wi 2667/2-3 & 2-4) to Oliver Wilhelm and Werner Sommer.

### Conflict of interest statement

The authors declare that the research was conducted in the absence of any commercial or financial relationships that could be construed as a potential conflict of interest.
